# X-ray absorption spectroscopy of lanmodulin-derived peptides bound to rare earth elements

**DOI:** 10.1107/S1600577525007726

**Published:** 2025-09-22

**Authors:** Adam Smerigan, Adam S. Hoffman, Jorge Perez-Aguilar, Rui Shi, Simon R. Bare

**Affiliations:** a Department of Chemical Engineering The Pennsylvania State University University Park PA USA; b Stanford Synchrotron Radiation Laboratory SLAC National Accelerator Laboratory Menlo Park CA94025 USA; c SUNCAT Center for Interface Science and Catalysis SLAC National Accelerator Laboratory Menlo Park CA94025 USA; d Institute of Energy and the Environment The Pennsylvania State University University Park PA USA; University of Essex, United Kingdom

**Keywords:** rare earth elements, lanthanides, EXAFS, XANES, lanmodulin, EDTA, peptide

## Abstract

A sustainable and robust supply chain of rare earth elements (REEs) is necessary to meet our consumer, national security and clean energy goals. However, current intra-REE separation technologies (*e.g.* solvent extraction) are costly and carry a heavy environmental burden. Therefore, the development of new aqueous based ligands that are selective for individual REEs will be integral in future REE production systems. To develop these ligands, an understanding of how ligand coordination structure relates to selectivity is imperative. We used X-ray absorption spectroscopy (XAS) to observe the local structure around four lanthanide (Ln) ions (La, Ce, Pr and Nd) complexed by water and several relevant chelating ligands [lanmodulin EF-hand 1 peptides (LanM1), ethyl­enedi­amine­tetra­acetic acid (EDTA), amino­tris­(methyl­ene­phospho­nic acid) (ATMP) and citric acid]. To collect these liquid-phase XAS spectra, we developed a new flow cell that prevents bubble interference and beam damage to the samples. In the X-ray absorption near-edge structure (XANES), we observed energy shifts in the white line, white line broadening and differences in the white line intensity of different Ln–ligand complexes between ligands. In the extended X-ray absorption fine structure (EXAFS), we distinguished differences in peak intensity and distance between coordinating ligands. Differences in the local coordination structure between Ln–LanM1 peptide complexes were more subtle compared with the other ligands (La–water, La–EDTA, La–ATMP and La–citric acid complexes). Further XANES and EXAFS studies, in combination with modelling and other techniques, could greatly improve our structural knowledge of how these aqueous ligands bind Ln ions and how they can be used to design more selective ligands for more efficient and sustainable REE separations.

## Abstract 2

This study uses X-ray absorption spectroscopy (XAS) to observe the structural and electronic differences of coordination complexes of lanthanides (La, Ce, Pr and Nd) bound by lanmodulin-derived peptides and other organic chelators in aqueous solution. The reported sensitivity of XAS for subtle changes in lanthanide coordination environment supports its use in designing more selective REE-binding ligands for separations.

## 1. Introduction

The rare earth elements (REEs) have many distinct properties that make them ideal for applications ranging from lasers and displays to catalysts and magnets (Jha, 2014 ▸). Therefore, these critical elements will be essential for reaching climate goals, ensuring national security and supplying modern technologies (IEA, 2021 ▸). The REEs are 17 metals including the lanthanide series, scandium and yttrium, and are primarily produced from mining operations in China, the United States of America and Australia. However, the global supply chain is primarily dominated by Chinese production and refinement, leading to significant supply chain instability (Guyaux, 2021 ▸; Tracy, 2020 ▸; Gosen *et al.*, 2019 ▸). Therefore, there is interest in investigating the use of unconventional feedstocks for REE production (Fritz *et al.*, 2023 ▸; Balaram, 2019 ▸; Binnemans & Jones, 2015 ▸). To utilize these unconventional feedstocks, a key technology is the intra-REE separation to obtain pure, individual REEs. Conventionally, this separation is performed using solvent extraction [or to a lesser extent ion exchange (Hu *et al.*, 2018 ▸)]. However, the solvent-extraction process uses toxic organic solvents and is inefficient, requiring as many as 200 stages to perform the separation (Vahidi & Zhao, 2017 ▸). Such an inefficient process has high costs and environmental impact, limiting its potential use with dilute unconventional feedstocks. Therefore, a new separation exhibiting higher selectivity between REEs is necessary to develop more sustainable and secure production of REEs in the US and abroad (Nash, 1999 ▸).

The driver of selectivity in these separations is the chelating ligands used to complex the REEs. Typical phospho­rus based extractants used in solvent extraction exhibit selectivities around 1.2 between adjacent REE ions, while newer diglycolamide ligands have reached selectivities of around 3 between adjacent REE ions (Xie *et al.*, 2014 ▸; ORNL, 2021 ▸). To avoid the use of toxic organic solvents in aqueous systems, sorptive technologies (*e.g.* ion exchange, chromatography, membrane adsorption) are increasingly popular for extracting REEs from leachates (Dong *et al.*, 2021 ▸; Dong *et al.*, 2024 ▸; Opare *et al.*, 2021 ▸; El Ouardi *et al.*, 2023 ▸; Ye *et al.*, 2023 ▸). These sorptive systems commonly use aqueous organic chelating ligands to adsorb REEs onto the solid phase. Common organic chelating molecules [*e.g.* ethyl­enedi­amine­tetra­acetic acid (EDTA)] have high affinity for REEs but limited selectivity between metals (Nash, 1999 ▸). Therefore, new bio-based ligands are being explored [*e.g.* lanthanide binding tag (Hatanaka *et al.*, 2020 ▸), Lanmodulin (LanM) protein (Deblonde *et al.*, 2020 ▸) and peptides of LanM EF-hand loop binding pockets (Verma *et al.*, 2024 ▸; Gutenthaler *et al.*, 2022 ▸)] with lower (micromolar) affinity but increased selectivity. These bio-based ligands (proteins and peptides) have unique amino acid sequences that dictate the selectivity. Both binding and non-binding amino acid residues have been shown to affect selectivity, with some studies identifying how changing these residues can influence water coordination in the first binding shell (Gutenthaler *et al.*, 2022 ▸; Sajeevan *et al.*, 2025 ▸; Liu *et al.*, 2021 ▸; Featherston *et al.*, 2021 ▸; Mattocks *et al.*, 2022 ▸). However, the structural basis for the selectivity of these bio-based ligands is still unclear.

Many different techniques have been used to determine the coordination structure around REEs in both the aqueous and the organic phase. However, each of these techniques has limitations. NMR best resolves the structure of the biomolecule’s backbone but has limited accuracy in determining the structure of the binding pocket (Fowler *et al.*, 2020 ▸). Molecular simulations can generate highly accurate structures but are limited by the computational expense of these calculations, especially for large biomolecules. X-ray diffraction (XRD) can also generate highly accurate structures of biomolecule coordination complexes with REEs but requires significant efforts to crystallize the coordination complex. Further, the solid crystal may not be representative of liquid phase structure due to the loss of outer sphere solvation and crystal packing forces (Levina *et al.*, 2005 ▸; MacNaughton *et al.*, 2006 ▸). To overcome some of these limitations, X-ray absorption spectroscopy (XAS) can be used to directly observe the local structure around REEs in the liquid phase (D’Angelo *et al.*, 2022 ▸; Migliorati *et al.*, 2021 ▸, 2018 ▸). The extended X-ray absorption fine structure (EXAFS) region of XAS spectra can provide detailed structural information including interatomic distances, disorder and coordination number of atoms around the REE atom. The X-ray absorption near edge structure (XANES) region contains information about the electronic structure around the absorbing REE atom. Therefore, XAS can be a critical tool for understanding the complex binding interactions near the REE atom, especially when coupled with some of the other techniques mentioned. XAS studies have primarily focused on the EXAFS of organic extractants (*e.g.* diglycolamides) to understand structure–function relationships. These studies found that steric repulsion, ligand strain and other outer sphere interactions are essential for enhancing selectivity in these ligands (Stamberga *et al.*, 2020 ▸; Ellis *et al.*, 2017 ▸; Brigham *et al.*, 2017 ▸). For biomolecules, no XAS studies have been performed, likely due to the difficulty of working with solubilized biomolecules and high-powered X-rays [*e.g.* beam damage, bubbling (Ekanayake *et al.*, 2024 ▸)]. However, there have been studies that determine the structure of LanM protein and LanM EF-hand loop peptides (abbreviated LanM1 for the EF-hand loop 1 peptide sequence) in aqueous solution using NMR (Cook *et al.*, 2019 ▸), infrared and fluorescence spectroscopy, and molecular simulations (Gutenthaler *et al.*, 2022 ▸; Sajeevan *et al.*, 2025 ▸; Liu *et al.*, 2021 ▸; Featherston *et al.*, 2021 ▸).

To supplement these works, we explore how XAS can be used to observe the local coordination structure around lanthanide ions bound by LanM1 peptide variants. We examine how changing non-binding residue 8 in LanM1 affects lanthanide coordination for four different lanthanides (La, Ce, Pr, Nd). We collected the *L*-edge XANES and EXAFS spectra for these lanthanides coordinated by each of 6 LanM1 variants. To collect these spectra, we developed a new flow cell to prevent bubble formation and beam damage to the sample. We were able to qualitatively observe how different peptide variants influenced the local coordination structure around the four different lanthanide ions. Future quantitative analysis of these EXAFS and XANES spectra, coupled with information from other techniques, can be used to develop an unprecedented structure–function understanding of Ln–biomolecule complexes.

## 2. Methods

### 2.1. Sample preparation

The lanthanide chlorides (all Ln had a valence of III) were purchased from Sigma–Aldrich at ≥99.9% trace-metal purity. The LanM1 peptides were purchased as lyophilized solids from GenScript with ≥95% purity. The N-terminal was acetyl­ated and the C-terminal was amidated to improve stability. The peptides were stored in a −50°C freezer except during sample preparation and were warmed to room temperature prior to weighing. For the peptide samples, first, lanthanide chloride was added to 1.5 ml of DI water (MilliQ water with a resistivity of 28 MΩ cm) to create a 0.05 *M* solution. Then an equimolar amount of peptide was added to the lanthanide (III) chloride solution to create a 0.05 *M* solution of lanthanide and peptide (one binding site per peptide molecule). The pH of the sample was adjusted to 6 using approximately 30 µl of a 7.5 *M* sodium hydroxide solution made from sodium hydroxide pellets from Sigma–Aldrich (≥99.9% purity). The pH was measured using a Hannah Instruments H18104 pH/ORD Meter with the AMANI-1000-L pH sensor and potential shifting device from Innovative Instruments. All samples were stored in the refrigerator at 4°C until use (for a maximum of 2 days). The hydro­chloric acid was purchased from Fisher Scientific at TraceMetal grade.

For samples containing EDTA, citric acid or amino­tris­(methyl­ene­phospho­nic acid) (ATMP), the samples were sealed inside polyethyl­ene X-ray fluorescence cups (SPEX SamplePrep 3577) using a 3.6 µm polyester (mylar) film window from Somar International. The EDTA, citric acid and ATMP were purchased from Sigma–Aldrich at ≥99%, ≥97% and ≥99.5% purities, respectively. Stock solutions of lanthanide (III) chloride and EDTA were made in MilliQ water (28 MΩ cm). The pH of each stock solution was adjusted to 6 using NaOH from Sigma–Aldrich at ≥99.9% purity. Samples were made with an equimolar concentration (0.05 *M*) of LnCl_3_ and ligand. The ATMP and citrate samples were observed to form a precipitant, while the EDTA samples did not precipitate at the desired pH.

### 2.2. Experimental flow cell materials and operation

A 07551-20 Masterflex L/S peristaltic pump was used to flow the sample through the tubing. The tubing used was L/S 13 Masterflex L/S Precision Pump Tubing. The sample was flowed through the tubing at a rate of 10 ml min^−1^. The sample was exposed to X-rays through a Kapton capillary (American Durafilm, 2.85 mm ID, 0.09 mm wall thickness) which was connected to the tubing using heat-shrink tubing. The use of heat-shrink tubing to connect the pump tubing and the Kapton capillary minimized the internal dead volume of the system associated with fittings.

To switch samples, first, the feed tubing was removed from the sample vial and the remaining sample was pumped into the sample vial. Next, DI water was flushed through the flow cell tubing for 1 min at 10 ml min^−1^. Following the water flush, the tubing was cleaned with 1 *M* hydro­chloric acid to remove any residue stuck to the tubing from the previous sample. After the acid wash, the tubing was flushed again with DI water for another minute. A scan was collected to confirm that there was no discernable absorption from the previous sample confirming that the flow cell was clean of the previous sample.

### 2.3. XAS data collection

The X-ray absorption spectra were collected at beamline 9-3 at the Stanford Synchrotron Radiation Lightsource within the SLAC National Accelerator Laboratory. Beamline 9-3 is a wiggler-based side station equipped with an Si(220), φ = 90°, double-bounce liquid-nitro­gen-cooled monochromator, and Rh-coated collimating and focusing mirrors located before and after the monochromator, respectively. The beamline 9-3 mirrors (pitched at 7.1 mrad) were operated in a 10 keV cutoff mode. With Si(220) crystals, the second harmonic is at 11.4 keV for Ce where there is far more flux at 11.4 keV and 5.7 keV at beamline 9-3. Therefore, the monochromator was detuned 40% to minimize the effect of harmonics. The flux of the X-ray is estimated to be 2 × 10^12^ photons s^−1^ in a spot size of 1 mm × 4 mm. XAS spectra were collected in continuous-scanning mode with the samples in a fluorescence geometry (sample at a 45° angle to the incident beam) using a PIPS diode with Soller slits with a 1 inch focal length to minimize signal from the scattered beam. Reference spectra of a manganese foil (calibrated to 6539.0 eV) or vanadium foil (calibrated to 5465.0 eV) were collected simultaneously as an internal energy standard by an off-axis photodiode. Nine spectra of each sample were collected to achieve a sufficient signal-to-noise ratio. Data from the *L*_3_-edge were used for samples containing La, Ce and Pr. However, the *L*_2_-edge was used for samples containing Nd due to several un-removable glitches in the Nd *L*_3_-edge spectrum. For the flow cell, the beam size was 1 mm (V) by 2.5 mm (H) which fits within the Kapton capillary (3 mm in diameter).

For the samples in the X-ray fluorescence cups, spectra were collected at the same beamline as specified above. The beam size was 1.0 mm (V) and 4 mm (H). XAS spectra were collected in step-scanning mode with the samples in a fluorescence geometry (sample at a 45° angle to the incident beam) using a PIPS diode with 10 cm Soller slits to minimize signal from the scattered beam. A spectrum of a manganese foil (calibrated to 6539.0 eV) was collected simultaneously as an internal energy standard by an off-axis photodiode. The Mn foil was additionally scanned without the sample to be used for energy alignment in the data processing. Two to four spectra of each sample were collected to achieve a sufficient signal-to-noise ratio.

The XAS data were processed using *Athena* from the *Demeter* software package (Ravel & Newville, 2005 ▸). The reference foils were calibrated to their edge positions using the peak of the first derivative (Henke *et al.*, 1993 ▸). The simultaneously collected reference spectra were then aligned to this calibrated reference foil spectrum to calibrate the sample data and determine the edge energy of the lanthanide. *Athena* was used to perform the energy calibration, normalization and EXAFS extraction.

## 3. Results

Across the three different *L*-edges, we collected the XANES and EXAFS of each coordination complex between six different LanM1 peptide variants and four different lanthanides. Furthermore, we collected spectra of samples containing only lanthanide ions coordinated by water for comparison to the peptide samples. Table 1 ▸ shows the six different LanM1 peptide variants examined including the amino acid sequence with the substitution on the eighth residue shown in bold, abbreviations, steric bulkiness and polarity. The LanM1-scrambled peptide is a random shuffle of the amino acids present in LanM1 that was assumed to disrupt the binding pocket. For visual clarity, we show a subset of the samples in many of the figures in the main text. The full set of spectra are included in the supporting information.

### 3.1. Flow cell performance

For liquid biological samples, liquid microjets and flow cells are commonly used to collect XAS data. Liquid microjets greatly reduce sample exposure to the beam but can be complicated to use and have several limitations (*e.g.* gas-phase background signal) (Smith & Saykally, 2017 ▸; Schwartz *et al.*, 2010 ▸; Uejio *et al.*, 2010 ▸). Flow cells have been used in many XAS applications (Smith & Saykally, 2017 ▸; Aziz *et al.*, 2009 ▸; Ostervold *et al.*, 2024 ▸; Deschner *et al.*, 2021 ▸) and generally consist of pumping liquid samples past an X-ray-transparent window(s) where the absorption signal can be measured using transmission or fluorescence geometry. Here, we developed a flow cell for collecting the fluorescence signal of an aqueous peptide solution (S7). This cell was designed to overcome two limitations that have been reported for XAS of biological samples: bubble from water radiolysis (Jay-Gerin, 2025 ▸; Mesu *et al.*, 2007 ▸; Xian *et al.*, 2024 ▸; Matsumoto *et al.*, 2022 ▸; Loh *et al.*, 2020 ▸) and beam damage to the sample (Ekanayake *et al.*, 2024 ▸; Ravelli & McSweeney, 2000 ▸; Carugo & Carugo, 2005 ▸). To overcome these limitations, the flow cell continuously flows sample across the path of the beam and deposits any bubbles formed to the top of the sample vial. Therefore, very little sample is exposed to the beam at any given time, reducing the amount of bubbles generated and beam damage to the sample. Further, the flow cell requires only 1.5 ml of sample, which keeps the cost per sample manageable at the relatively high concentrations of peptide used here (0.05 *M*). Therefore, this cell can be used for collecting high-quality XAS spectra of aqueous peptide complexes.

**Figure 1:**
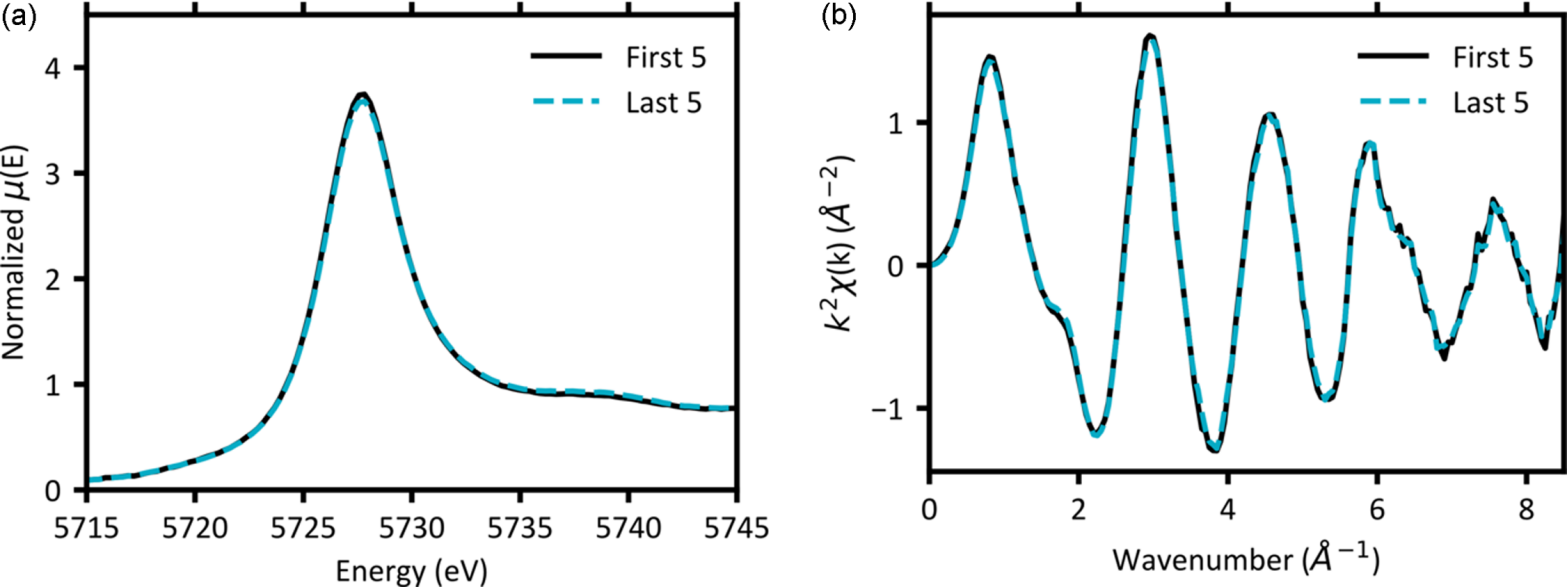
Ce *L*_3_-edge (*a*) XANES and (*b*) *k*-space of the Ce–water sample collected in nine continuous scans of 92 s each. Merged scans of the first and last five scans show indicate beam damage over 13.8 min of exposure to the beam.

To quantify beam damage on the samples, we analysed both the white line intensity and the EXAFS data in *k*-space over the nine scans for each sample. The total scan time for each sample was 13.8 min for nine replicates in continuous scan collection mode. The most damaged sample was the Ce sample in water, which had a decrease in white line intensity of 4% over the nine scans. To visualize this damage, Fig. 1 ▸ shows the first and last five XANES and *k*-space spectra collected for the Ce and water sample. For the other samples, any spectral changes resulting from X-ray induced damage could not be distinguished from the noise in the data. Additionally, there was no observable distortion of the signal due to bubble interference like we observed in the XAS spectra collected for the samples in the XRF cups (S2). The resulting spectra are of high quality (up to *k* of around 8.5 Å^−1^). The flow cell enabled efficient collection of XAS data across multiple lanthanide*L*-edges without significant beam damage making efficient use of high-demand beam time.

### 3.2. XANES of Ln–peptide complexes

**Figure 2:**
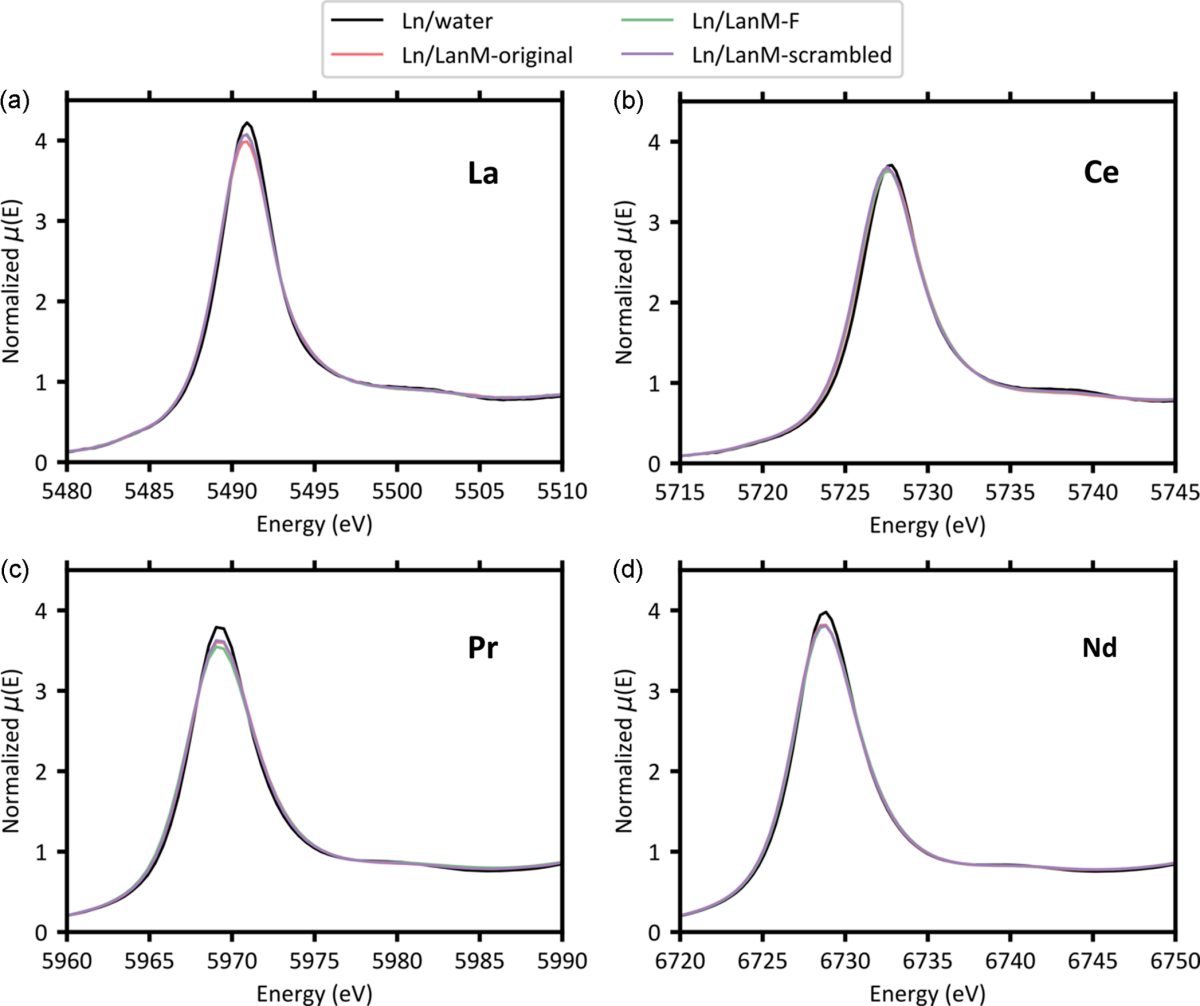
Normalized XANES spectra of water and three peptide variants complexed with (*a*) La (*L*_3_-edge), (*b*) Ce (*L*_3_-edge), (*c*) Pr (*L*_3_-edge) and (*d*) Nd (*L*_2_-edge).

**Figure 3:**
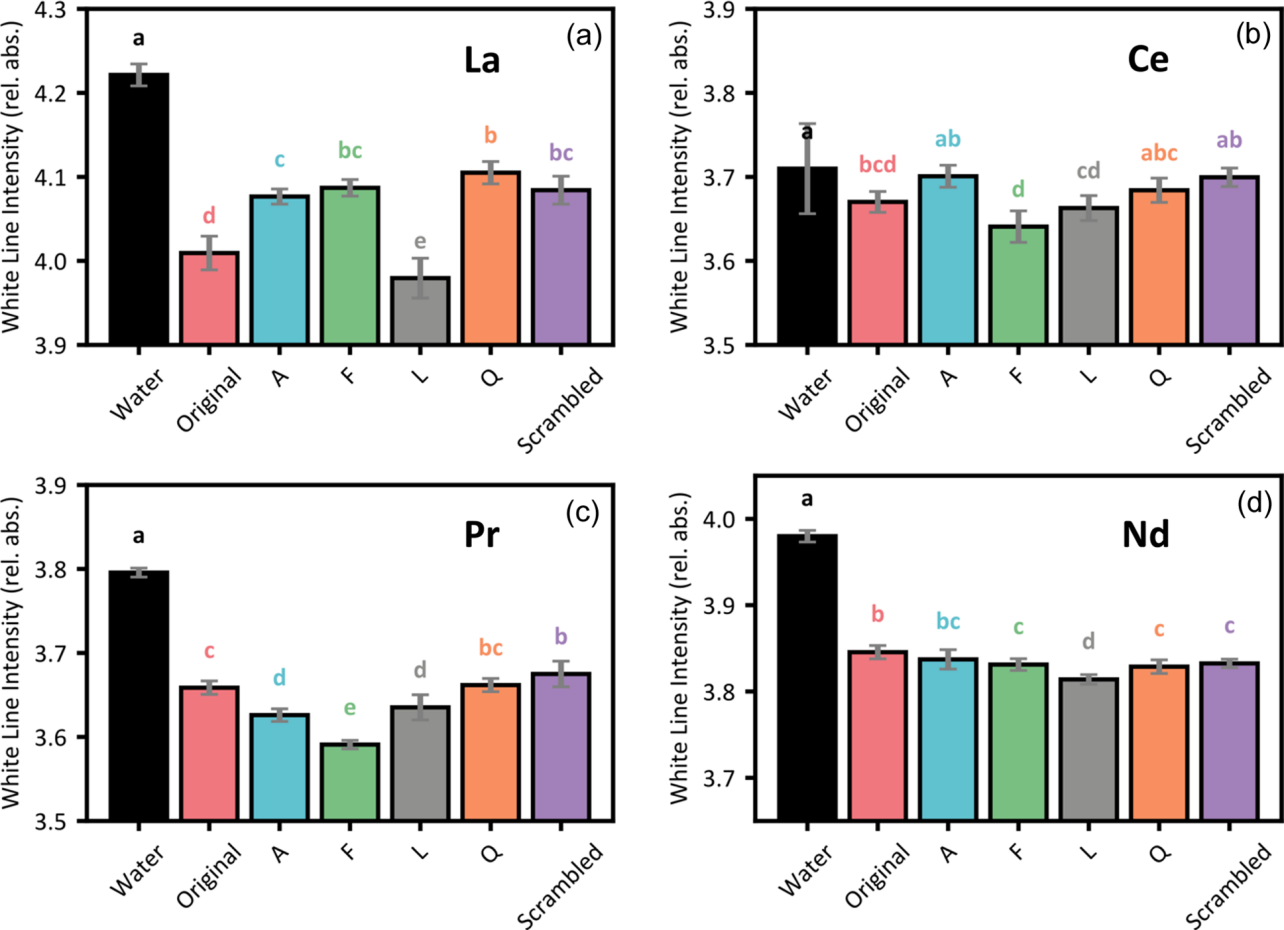
White line intensities of XANES spectra for water and the six peptide variants (*x* axis) complexed with (*a*) La (*L*_3_-edge), (*b*) Ce (*L*_3_-edge), (*c*) Pr (*L*_3_-edge) and (*d*) Nd (*L*_2_-edge). Error bars represent the standard deviation of the nine scans taken for each sample. Samples that share a letter in the coloured letter codes above the bars are not statistically different (*p* < 0.05).

The XANES spectra of three LanM1 variants complexed with La, Ce, Pr and Nd are shown in Fig. 2 ▸. These XANES spectra show that peptide coordination of the lanthanide ions is visually distinct in three main ways compared with those from Ln–water complexes. First, the white line energy shifts to the left for Ln–peptide samples (−0.1 eV for La, −0.2 eV for Ce, 0.0 eV for Pr and −0.2 eV for Nd). The specific peptide variant in the sample does not seem to influence this white line shift. Second, the Ln–peptide XANES have a slightly broader white line peak compared with Ln–water samples. Further, the post-edge oscillations are diminished for Ln–peptide samples. Similar to the white line shifts, between the peptide variants, there are no distinguishable differences in white line broadening and post-edge features. Third, the intensity of the white line decreases by approximately 4% when La, Pr and Nd are complexed by peptides rather than only water. However, there is only a decrease of around 1% for Ce–peptide complexes. Fig. 3 ▸ shows whether there is a statistically significant difference between the white line intensities of different Ln–peptide complexes. In general, there was more variation in the white line intensities of La and Pr complexes, compared with Ce and Nd. Additionally, the relative intensity rankings of peptide complexes differ between the lanthanides. For example, the La–peptide complex with the lowest intensity (LanM1-L) does not remain the lowest when other lanthanide metals are used. This suggests that each LanM1 variant exhibits distinct local electronic interactions with different lanthanide ions. Overall, uncertainties are similar between all Ln–ligand complexes. However, we assign the larger uncertainty in the Ce–water spectra as a result of the beam damage observed in this sample.

### 3.3. EXAFS of Ln–peptide complexes

**Figure 4:**
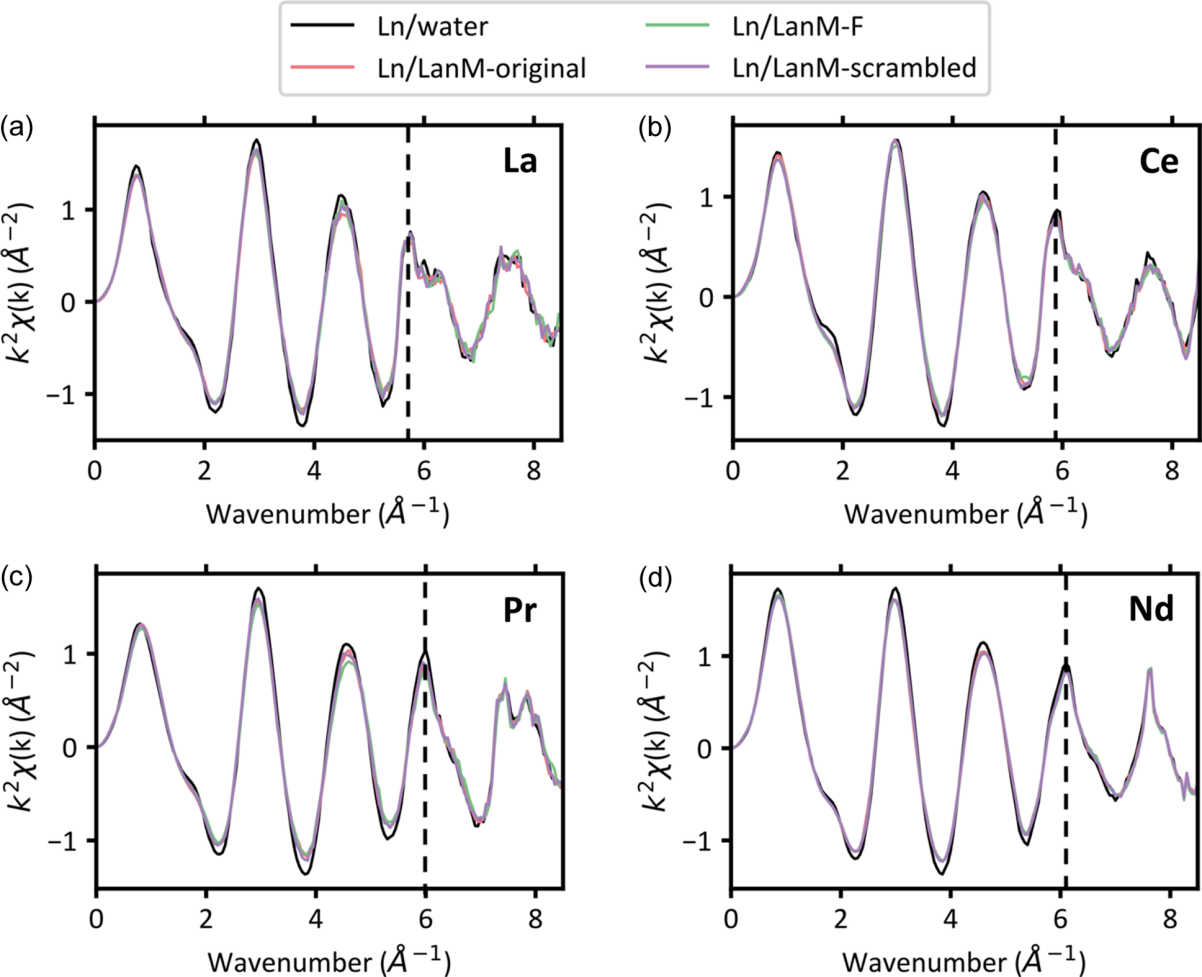
Measured Ln *L*-edge *k*-space spectra (*k*^2^-weighted) of water and the three of the peptide variants complexed with (*a*) La (*L*_3_-edge), (*b*) Ce (*L*_3_-edge), (*c*) Pr (*L*_3_-edge) and (*d*) Nd (*L*_2_-edge). The MEEs are indicated by the vertical dashed line.

The *k*-space data of three of the LanM1 variants complexed with La, Ce, Pr and Nd are shown in Fig. 4 ▸. In these spectra, there is a visible multielectron excitation (MEE) for all four lanthanides. This MEE is an unexpected feature of Ln *L*-edge XAS due to an additional excitation from the 2*p*, 4*d* → 5*d*^2^ transitions for the *L*_3_- and *L*_2_-edges and 2*s*, 4*d* → 6*p*, 5*d* for the *L*_1_-edge (Ohta *et al.*, 2008 ▸; Solera *et al.*, 1995 ▸). This MEE feature is strongest for La and appears at approximately 5.9 Å^−1^ before shifting higher in wavenumber (6.1 Å^−1^ for Nd) and diminishing in intensity across the lanthanide series. Samples with more disorder, like the aqueous samples here, generally have more noticeable MEEs compared with crystalline samples (Solera *et al.*, 1995 ▸). These MEEs have a modest influence on the results from EXAFS modelling (Ohta *et al.*, 2008 ▸; Solera *et al.*, 1995 ▸; Chaboy *et al.*, 1994 ▸) and can be removed in common programs used for data preprocessing (Ravel & Newville, 2005 ▸). Additionally, the *L*_3_- and *L*_2_-edge EXAFS is restricted to approximately 8.5 Å due to the presence of the next *L*-edge (Smerigan *et al.*, 2024 ▸). We found that the *L*_1_-edge did not have enough intensity to be useful for EXAFS analysis. However, the *L*_1_-edge XANES could be useful for examining a different electronic transition than that of the *L*_3_- and *L*_2_-edges.

**Figure 5:**
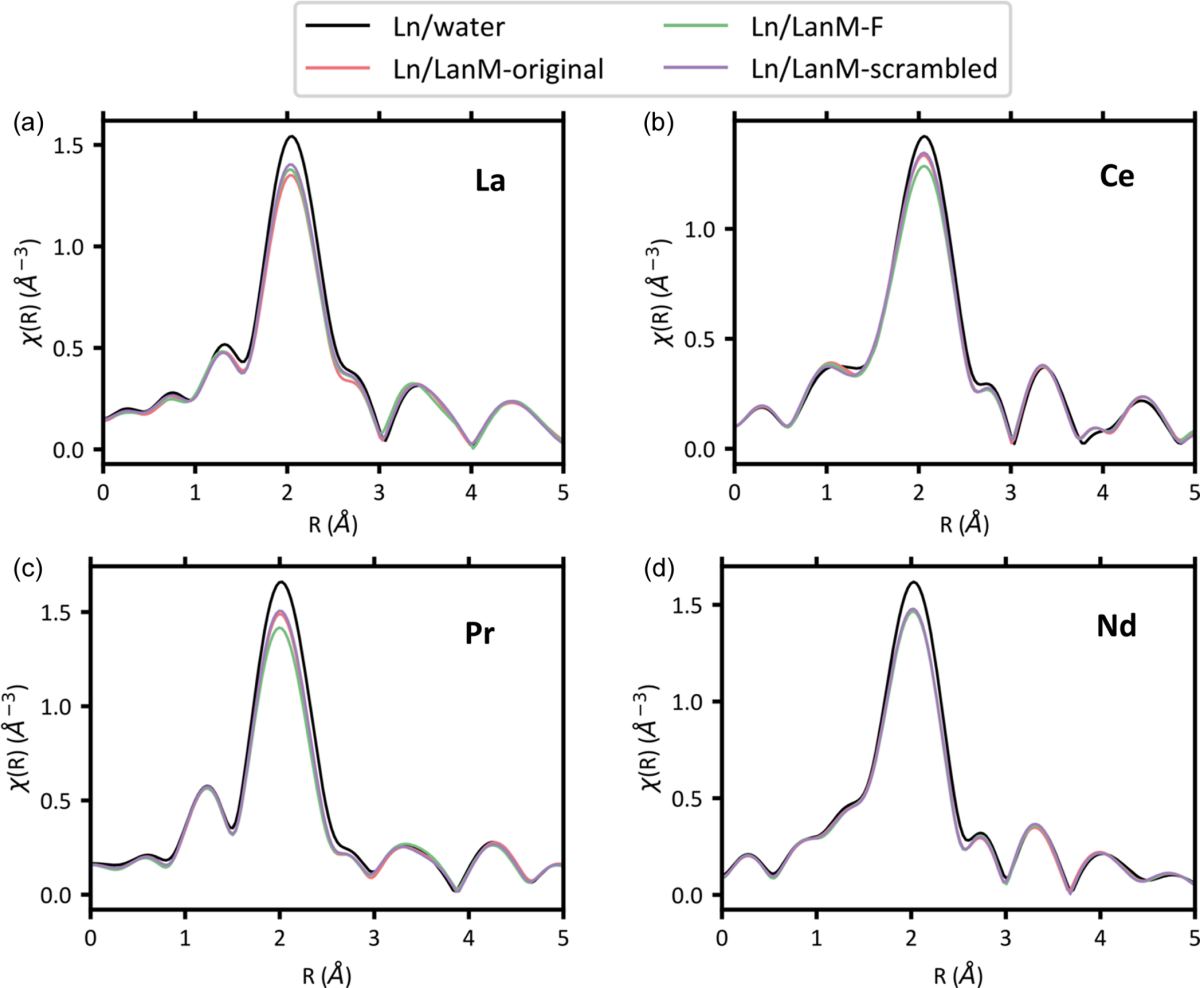
Measured Ln *L*-edge *R*-space spectra (*k*^2^-weighted) of water and three peptide variants complexed with (*a*) La (*L*_3_-edge, *k*-range: 2.53–8.5 Å^−1^), (*b*) Ce (*L*_3_-edge,*k*-range: 2.53–8.5 Å^−1^), (*c*) Pr (*L*_3_-edge, *k*-range: 2.53–8.5 Å^−1^) and (*d*) Nd (*L*_2_-edge, *k*-range: 2.53–8.5 Å^−1^).

The *R*-space of three LanM1 variants complexed with La, Ce, Pr and Nd are shown in Fig. 5 ▸. Similarly to the XANES results, the EXAFS of the peptide samples are distinguishable from the samples containing only lanthanide ions complexed by water. Unlike Ln–protein complexes that contain only two coordinated waters in the inner sphere (Alasadi *et al.*, 2024 ▸), these Ln–peptide complexes have been shown to coordinate up to five waters with micromolar affinity ensuring nearly complete binding in solution (Verma *et al.*, 2024 ▸; Gutenthaler *et al.*, 2022 ▸). This higher coordination of waters in the Ln–peptide complex is supported by the similarity to the EXAFS of Ln–water in Figs. 4 ▸, 5 ▸ and 6 ▸.

If there is any difference between the EXAFS of the peptide variants, it is present in very subtle changes in the intensity of the first peak around 2 Å and the intensity and radial distance of the second peak around 3.5 Å. Specifically, LanM1-F appears the most different from other peptide variants when complexed with La and Pr. We have provided the *k*-space and *R*-space for complexes of all four Lns with six LanM1 variants and water in the supporting information.

### 3.4. Comparison of lanthanum coordination with other chelating ligands

**Figure 6:**
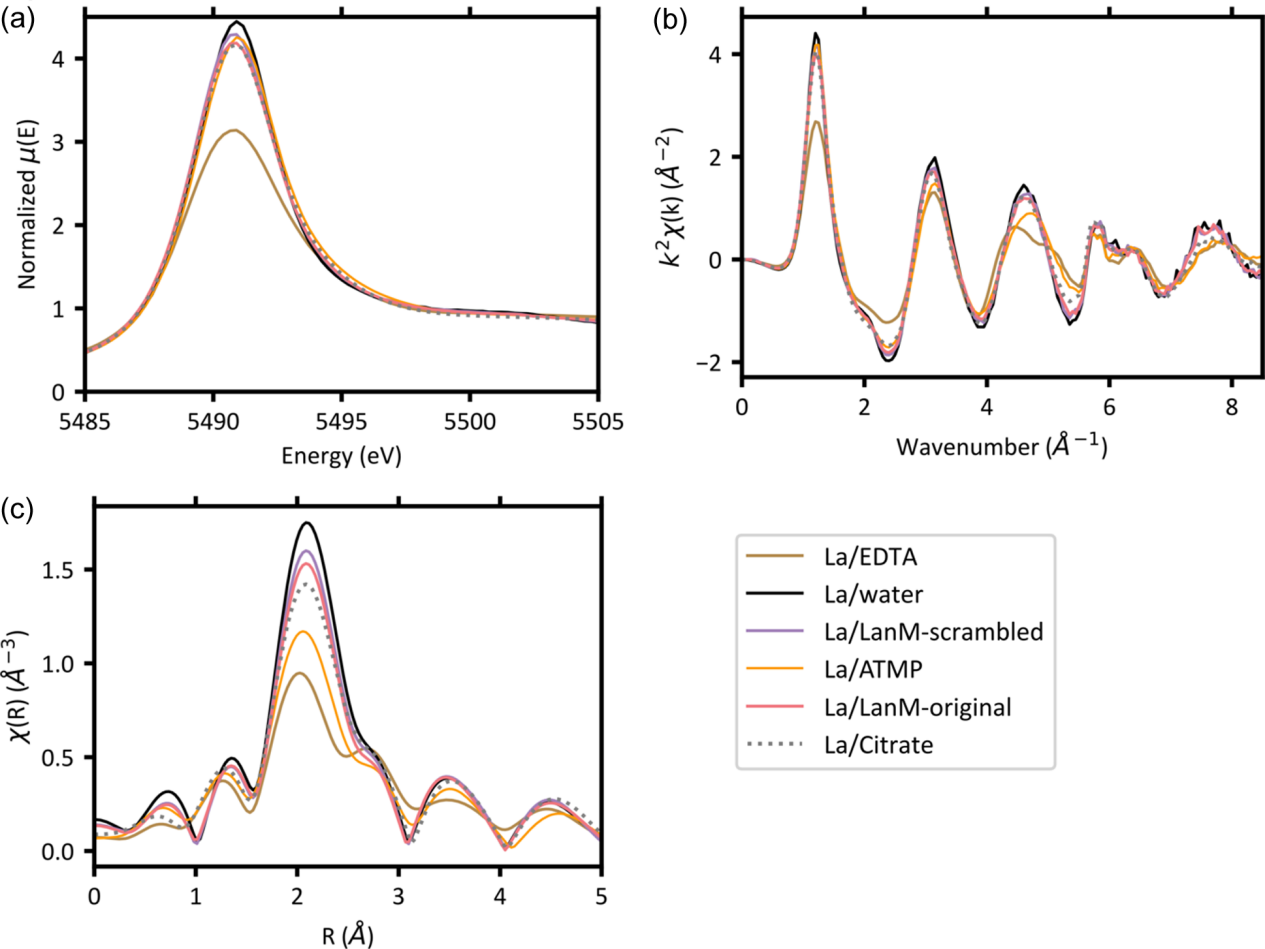
La *L*_3_-edge (*a*) XANES, (*b*) *k*-space and (*c*) *R*-space (*k*-range: 2.53–8.5 Å^−1^) spectra (*k*^2^-weighted) of La coordination complexes with water, LanM1 peptide, citric acid, ATMP and EDTA.

In addition to La coordination by LanM1 peptide variants, we compared La complexation by other chelating ligands with coordinating carb­oxy­lic oxygen atoms (citric acid, EDTA and ATMP). Fig. 6 ▸ shows the XANES, *k*-space and *R*-space of each of these ligands with La. In Fig. 6 ▸(*a*), the XANES spectra show clear differences between ligands through shifts in white line energy and intensity, where complexes with EDTA and peptide ligands shift lower in energy compared with water and ATMP shifts higher. In Figs. 6 ▸(*b*) and 6 ▸(*c*), the EXAFS spectra of the different Ln–ligand complexes have differences in phase and intensity. Of note, the *k*-space spectra of La–EDTA and La–ATMP complexes exhibit a phase shift around the wavenumber 4.5 Å^−1^, with the La–EDTA sample also displaying peak splitting [Fig. 6 ▸(*b*)]. In Figs. 6 ▸(*b*) and 6 ▸(*c*), the *k*-space and *R*-space spectra of La–water complexes have the largest peak intensity, which decreases for the chelating ligands. Further, this decrease in peak intensity appears correlated with shorter scattering distances for the first peak in *R*-space at around 2 Å [Fig. 6 ▸(*c*)]. We observed that the differences between the LanM1 peptide variants are smaller than the differences between LanM1 variants and the other chelating ligands (EDTA, citric acid and ATMP). Overall, XAS was effective in distinguishing between chelating ligands despite the ligands having similar binding groups (carb­oxy­lic oxygens), indicating the sensitivity of XAS for the distance and disorder of these coordinating atoms around Ln elements.

## 4. Discussion

From these results, we observed that XAS is sensitive to the local chemical environment around Ln ions. In the EXAFS spectra we observed that chelators with the highest affinity (EDTA and ATMP) have the shortest scattering distances and lowest intensity in the first peak like in other studies (Licup *et al.*, 2024 ▸). This trend is also observed in the peptide variants, as the LanM1-scrambled peptide has the most ‘water-like’ spectra due to the disruption of the binding pocket and higher coordination of water molecules (Verma *et al.*, 2024 ▸). Since subtle changes in inner-sphere binding are observed in the EXAFS, EXAFS modelling can be pursued to further understand structural differences between Ln–LanM1 peptide complexes. However, with the limited information content of the Ln *L*-edges, differences observed from EXAFS modelling may not be statistically significant. Therefore, other tools could be leveraged – such as molecular simulations or luminescence studies – to provide additional information for use in the EXAFS modelling to reduce the number of fitting parameters by setting physically relevant constraints.

In the XANES, we observed statistically significant differences in the white line intensities of the Ln–ligand complexes. However, for the LanM1 peptide variants, these differences are small and difficult to discern due to limited resolution. Therefore, higher-resolution measurements may be required using a high-energy resolution fluorescence detected (HERFD) XANES spectrometer-equipped beamline to get more detailed information about the electronic environment around the lanthanide atom. Coupling these HERFD–XANES spectra with density functional theory simulations could help identify electronic transitions unique to specific peptide variants (Zasimov *et al.*, 2022 ▸; Asakura *et al.*, 2019 ▸). Further, HERFD has been used to extend the useful EXAFS range for the *L*-edges of Ln elements (Glatzel *et al.*, 2005 ▸; Manceau *et al.*, 2024 ▸), which can greatly improve the ability to identify the number and organization of coordinating atoms through EXAFS modelling.

Additionally, we examined LanM1 peptide variants with changes limited to the non-binding, eighth residue. Since XAS is only sensitive to the local environment around the lanthanide ion (<5 Å), it is perhaps not surprising that a change to this non-binding residue located distantly from the lanthanide atom would have limited influence on XAS spectra. Therefore, it is likely that making more radical changes to peptide chemistry including changes to the binding ‘teeth’, that would be directly observed by XAS, would yield more statistically significant conclusions. Based on the results we gathered using this new flow cell, continuing with EXAFS and XANES modelling has the opportunity to provide insight into the chemistry of lanthanide chelation. As has been done in similar systems (Stamberga *et al.*, 2020 ▸; Ellis *et al.*, 2017 ▸), XAS could be used to help identify drivers of ligand selectivity between lanthanides in the aqueous phase. For example, if peptides exhibit little structural difference between lanthanide elements but large differences in affinity, then we could hypothesize that entropic differences (*e.g.* conformational changes on binding and water reorganization) dominate over the direct binding interaction. Similarly, with molecular modelling, we could observe how inner-sphere water coordination, which has been identified as important to selectivity, changes between ligands and lanthanides as has been done in similar systems (Shiery *et al.*, 2021 ▸; D’Angelo *et al.*, 2010 ▸; Ellis *et al.*, 2017 ▸; Stamberga *et al.*, 2020 ▸; Smerigan *et al.*, 2023 ▸).

## 5. Conclusions

This study identifies XAS as another tool to be used in combination with other techniques [*e.g.* molecular simulations, isothermal titration calorimetry, quartz crystal microbalance, fluorescence spectroscopy, NMR and circular dichroism] in understanding the structural basis of selectivity for lanthanide-binding chelating ligands. We introduced a new flow cell that was used for efficient collection of XANES and EXAFS spectra of lanthanide–LanM1 peptide variant coordination complexes in aqueous solution without issues from bubbles or beam damage. Further, this flow cell used low volumes of sample (1.5 ml), which was convenient for liquid samples containing expensive biomolecules and metals. XANES spectra showed shifts in edge energy between lanthanides coordinated by water, peptides and other chelating ligands. Further, the white line intensity changed between ligands and peptide variants across the four lanthanides examined (La, Ce, Pr and Nd). The rank-order of these white line intensities changed depending on the Ln metal indicating REE specific changes in coordination. In the EXAFS, we observed differences in peak intensity and scattering distance depending on the coordinating ligand. However, limited resolution and information content makes subtle differences more difficult to analyse, especially for the LanM1 peptide variants. Therefore, future work should focus on improving resolution (*e.g.* HERFD-XAS), examining other peptide variants, quantitative modelling of the XAS spectra and utilizing other tools (*e.g.* molecular simulations) to identify physical constraints for use in modelling the structure. From this increased structural knowledge, we can develop more selective ligands for intra-REE separations to promote REE supply chain security and meet the demand for green energy technologies.

## 6. Related literature

The following reference is cited in the supporting information: Calvin (2013 ▸).

## Supplementary Material

Supporting figures and tables. DOI: 10.1107/S1600577525007726/rv5196sup1.pdf

## Figures and Tables

**Table 1:** Peptide variants used in this study and their bulkiness and charge Variants are specified by the amino acid in the eighth residue of the peptide.

Amino acid sequence	Lanmodulin EF-hand 1 variant abbreviations	Bulkiness (No. of carbons in *R*-group)	Polarity/charge
DPDKDGT**I**DLKE	LanM1-original	4	Hydro­phobic
DPDKDGT**A**DLKE	LanM1-A	1	Hydro­phobic
DPDKDGT**F**DLKE	LanM1-F	7	Hydro­phobic
DPDKDGT**L**DLKE	LanM1-L	4	Hydro­phobic
DPDKDGT**Q**DLKE	LanM1-Q	3	Polar/uncharged
KGEDDDKTLDIP	LanM1-scrambled	4	Hydro­phobic

## Data Availability

Data made available upon request.
