# Supplementary material for: X-ray absorption spectroscopy of lanmodulin-derived peptides bound to rare earth elements
Source: J Synchrotron Radiat. 2025 Sep 22;32(Pt 6):1421–30. doi: 10.1107/S1600577525007726 (PMC12591073; doi:10.1107/S1600577525007726)
Supplement: Supplementary file 1 [file s-32-01421-sup1.pdf]

JOURNAL OF SYNCHROTRON RADIATION

Volume 32 (2025)

Supporting information for article:

X-ray absorption spectroscopy of lanmodulin-derived peptides bound to rare earth elements

Adam Smerigan, Adam S. Hoffman, Jorge Perez-Aguilar and Simon R. Bare

Table of Contents

S1. Sample Preparation 2

S2. XAS Spectra Exemplifying the Bubbling Issue 3

S3. XAS Spectra of La/ligand Complexes 4

S4. XAS Spectra of Ce/ligand Complexes 6

S5. XAS Spectra of Pr/ligand Complexes 8

S6. XAS Spectra of Nd/ligand Complexes 10

S7. Flow Cell 12

S8. XAS Preprocessing Details 16

S9. R-space spectra from 0-10 Å for Figure 5 and Figure 6c 18

S10. First-shell EXAFS Fit of La/LanM-orig complex 21

S11. References 22

S1. Sample Preparation

Peptide Variant	Lanthanide			
	La	Ce	Pr	Nd
Original	6.82	5.54	6.09	5.72
L	6.75	6.18	6.11	6.07
F	6.28	6.08	6.2	5.85
A	6.14	5.9	6.05	5.9
Q	6.5	6.1	5.6	5.91
Random	6.1	5.81	5.8	5.73

Table S1 The pH of the LanM1 peptide samples measured by XAS.

S2. XAS Spectra Exemplifying the Bubbling Issue

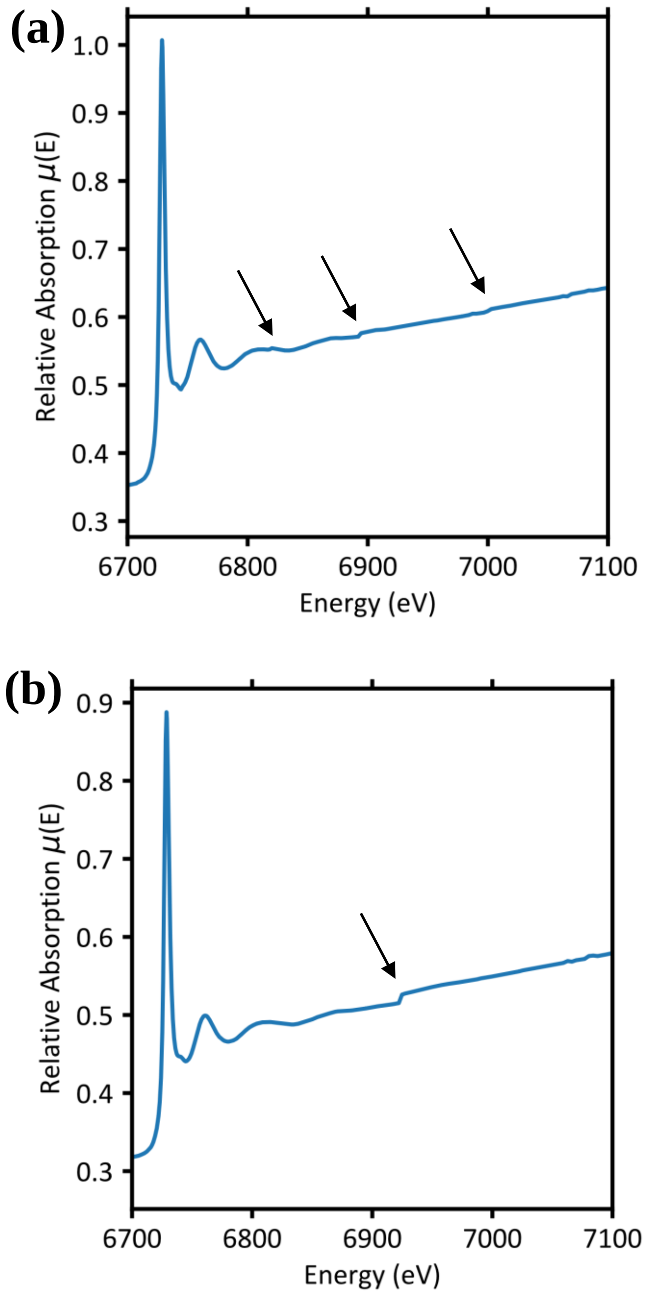

Figure S2 The raw L_2-edge XAS spectra of Nd complexed with ATMP where we observed interruptions from **(a)** small bubbles and **(b)** a large bubble. The disturbances due to bubbles are marked with an arrow. No glitches were observed at these energies and the location of these disturbances changed over time as bubbles formed.

S3. XAS Spectra of La/ligand Complexes

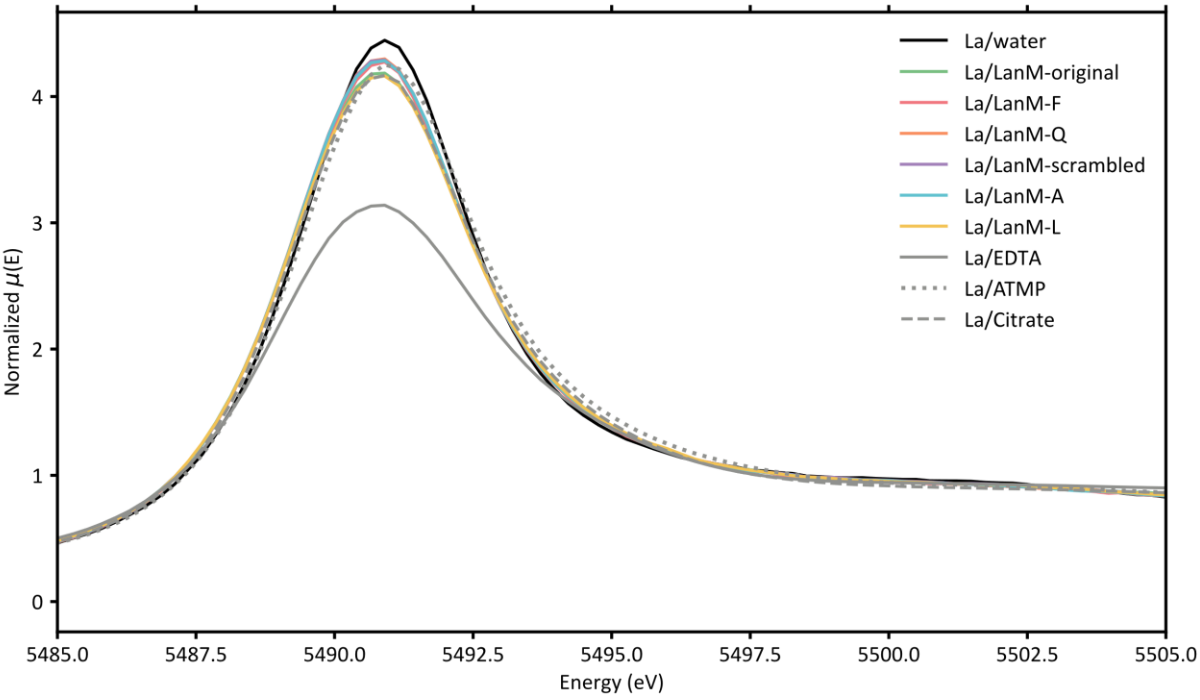

Figure S3 The XANES of water, the six peptide variants, EDTA, ATMP, and citric acid complexed with La (L3-edge).

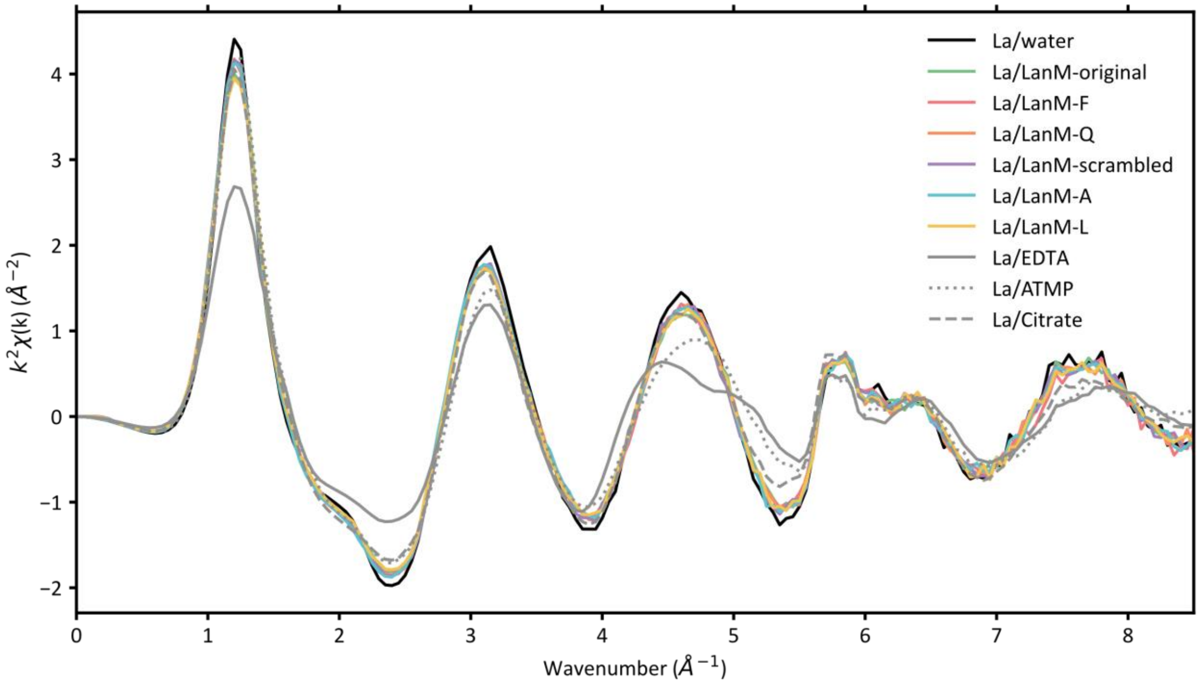

Figure S4 The k-space of water, the six peptide variants, EDTA, ATMP, and citric acid complexed with La (L3-edge).

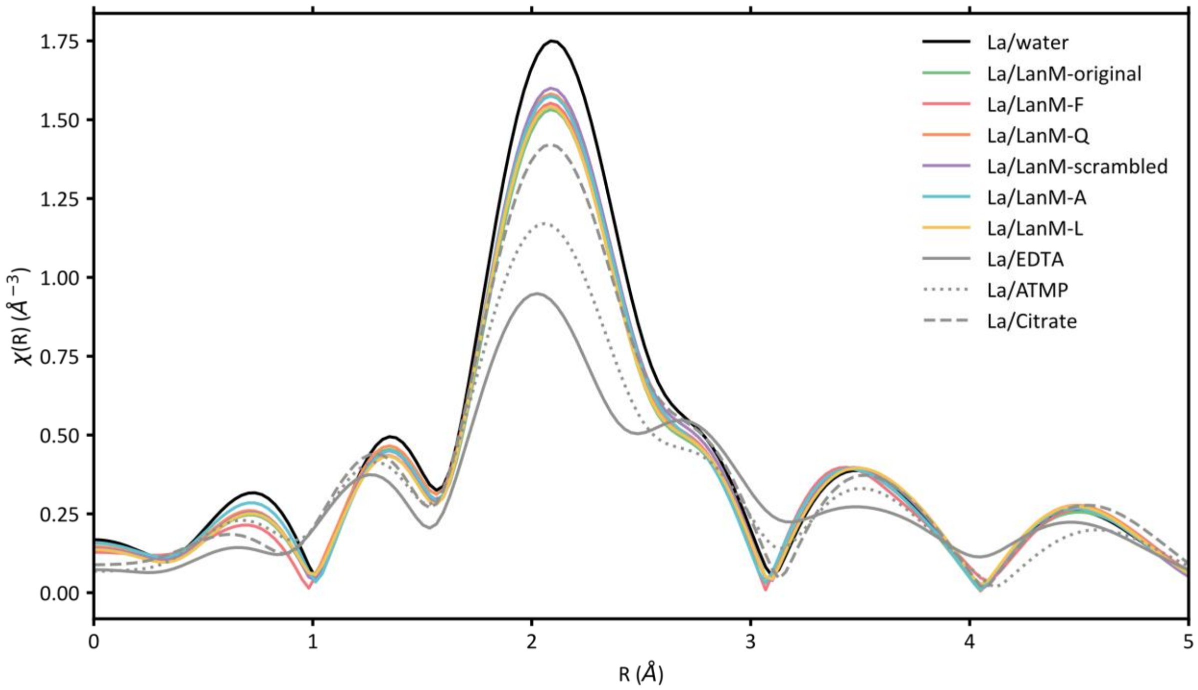

Figure S5 The R-space of water, the six peptide variants, EDTA, ATMP, and citric acid complexed with La (L3-edge and k-range: 2.53-8.5 Å^{-1}).

S4. XAS Spectra of Ce/ligand Complexes

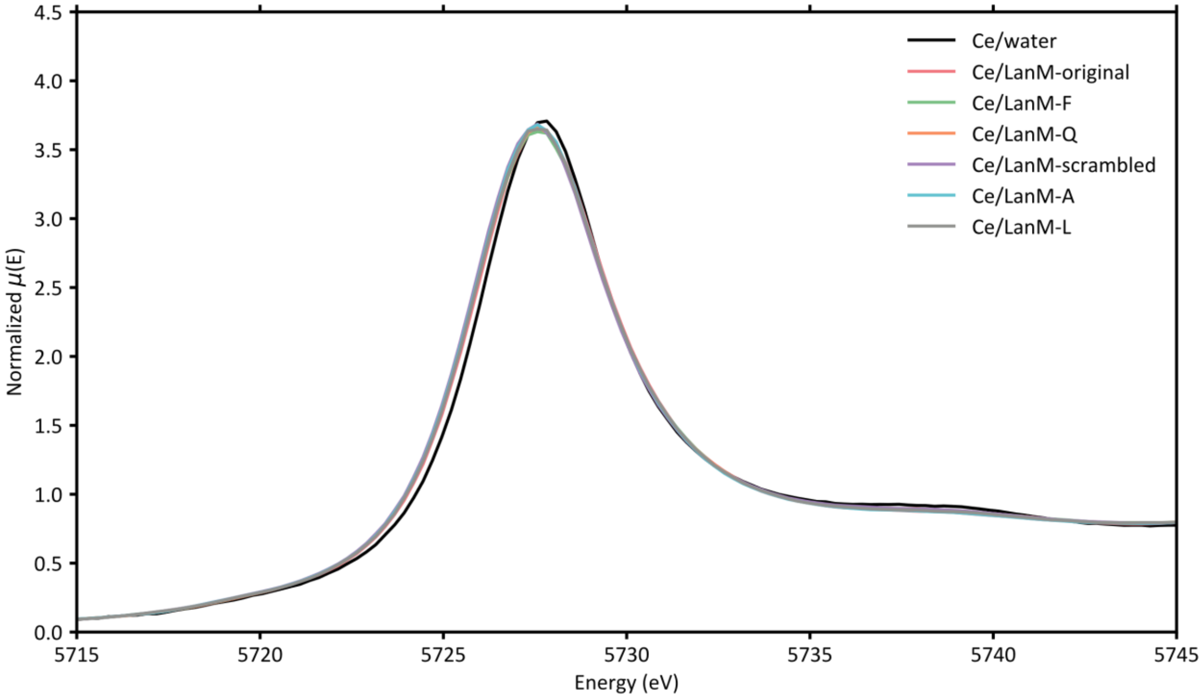

Figure S6 The XANES of water and the six peptide variants complexed with Ce (L3-edge).

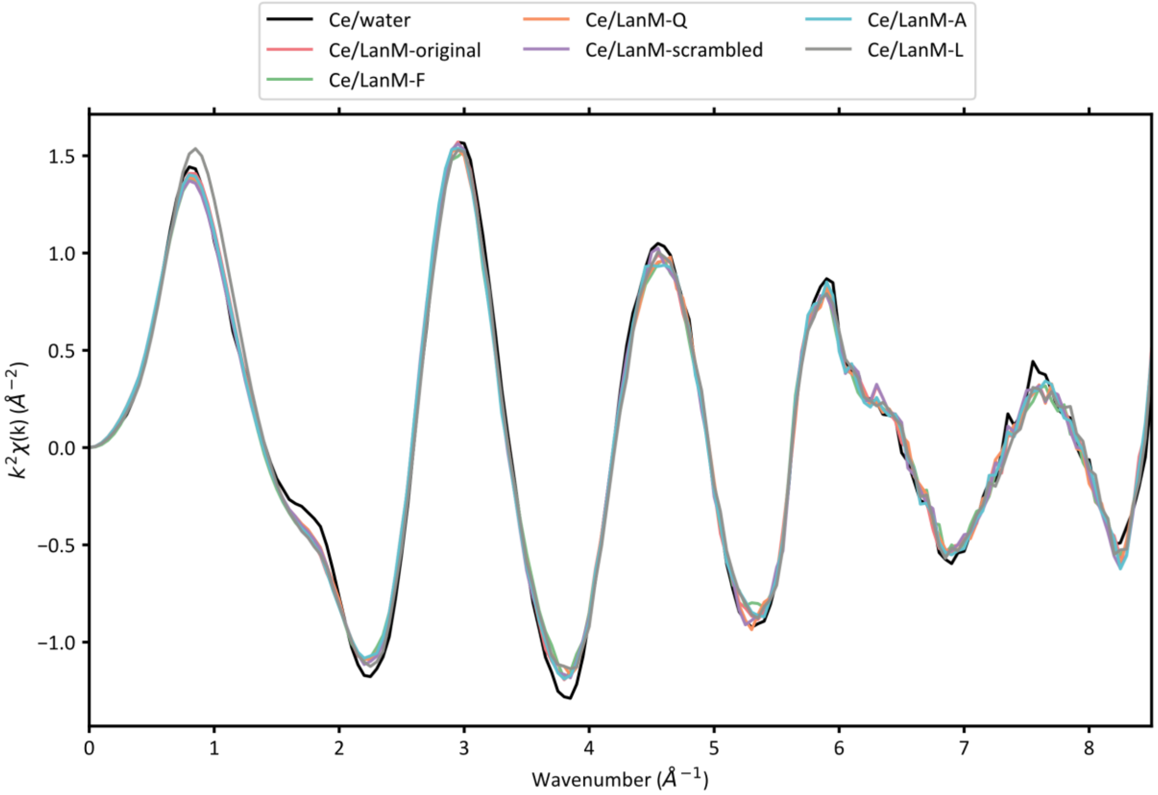

Figure S7 Figure S1: The k-space of water and the six peptide variants complexed with Ce (L3-edge).

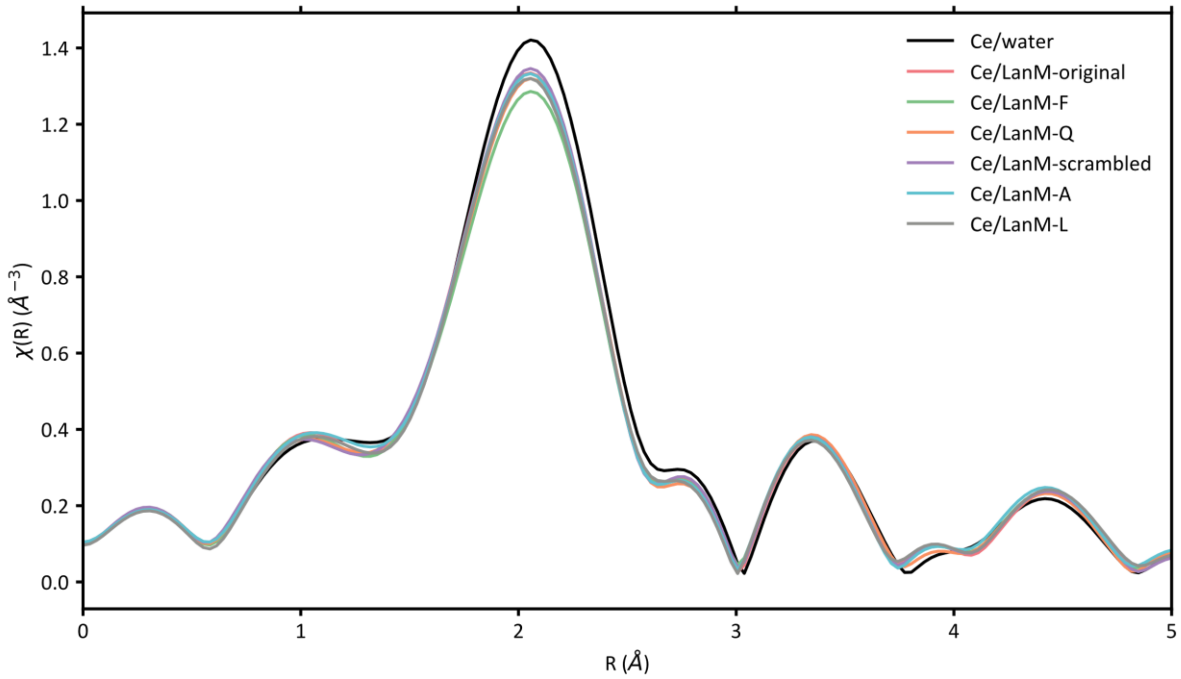

Figure S8 The R-space of water and the six peptide variants complexed with Ce (L3-edge and k-range: 2.53-8.5 Å-1).

S5. XAS Spectra of Pr/ligand Complexes

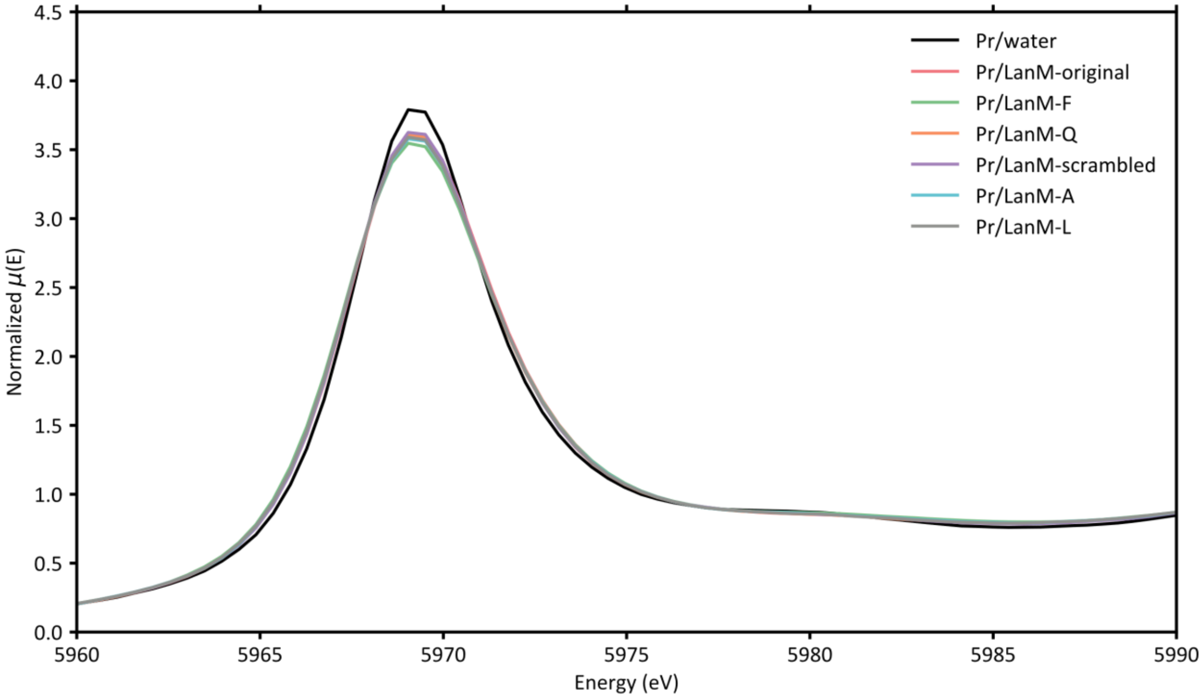

Figure S9 The XANES of water and the six peptide variants complexed with Pr (L3-edge).

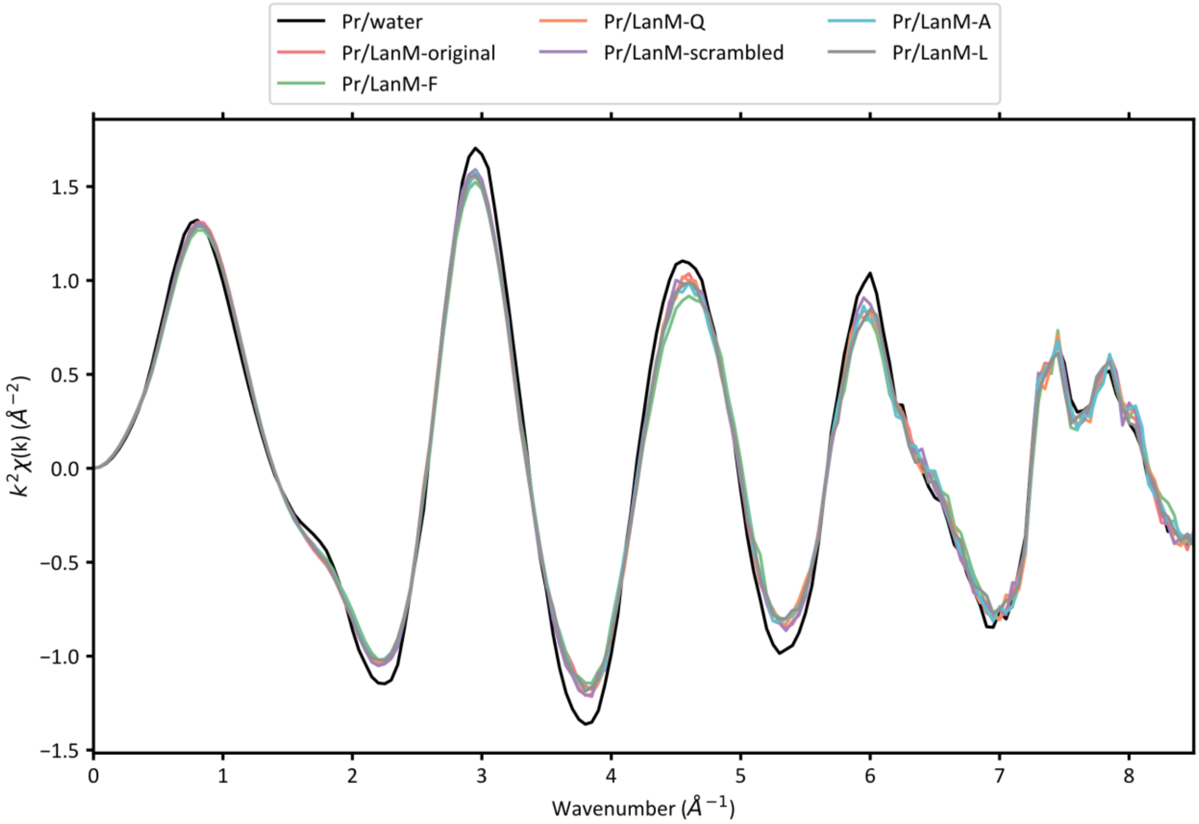

Figure S10 The k-space of water and the six peptide variants complexed with Pr (L3-edge).

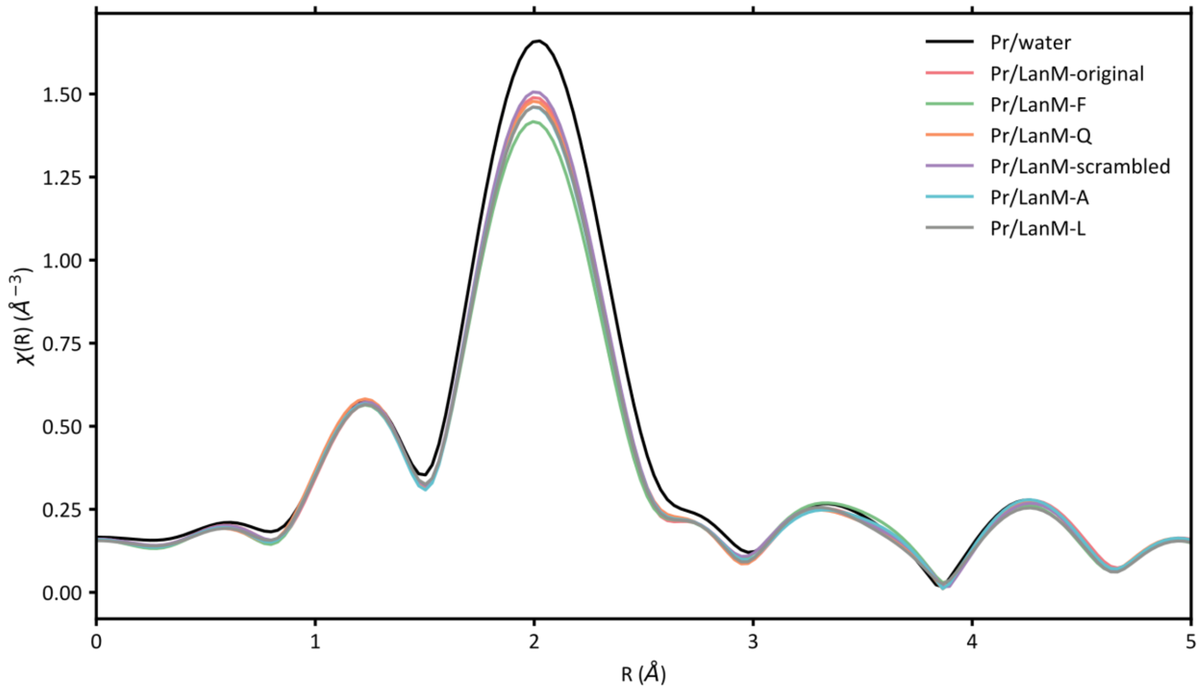

Figure S11 The R-space of water and the six peptide variants complexed with Pr (L3-edge and k-range: 2.53-8.5 Å-1).

S6. XAS Spectra of Nd/ligand Complexes

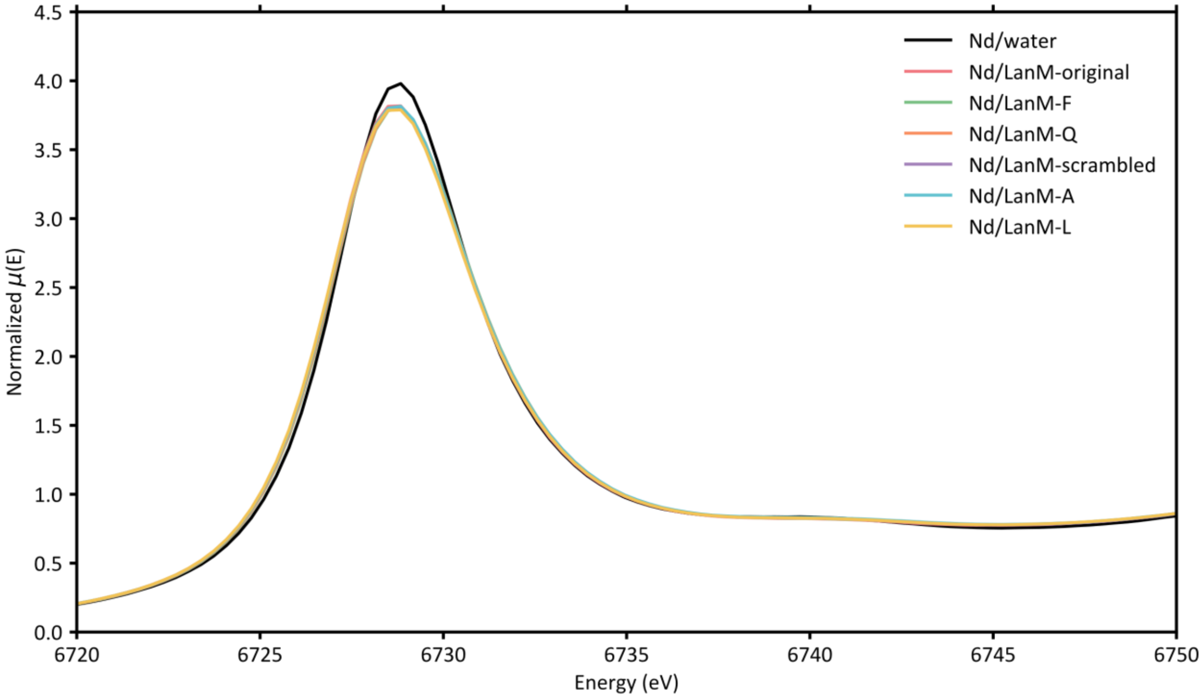

Figure S12 The XANES of water and the six peptide variants complexed with Nd (L2-edge).

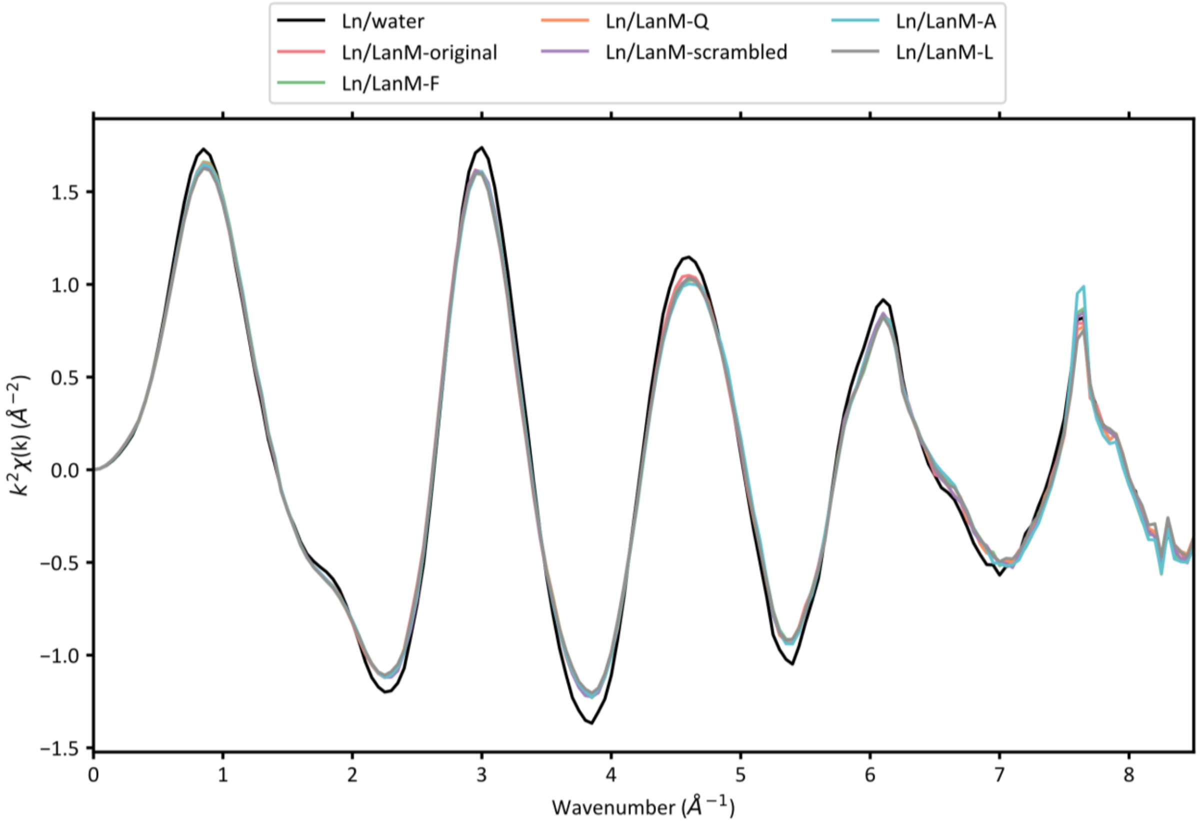

Figure S13 The k-space of water and the six peptide variants complexed with Nd (L2-edge).

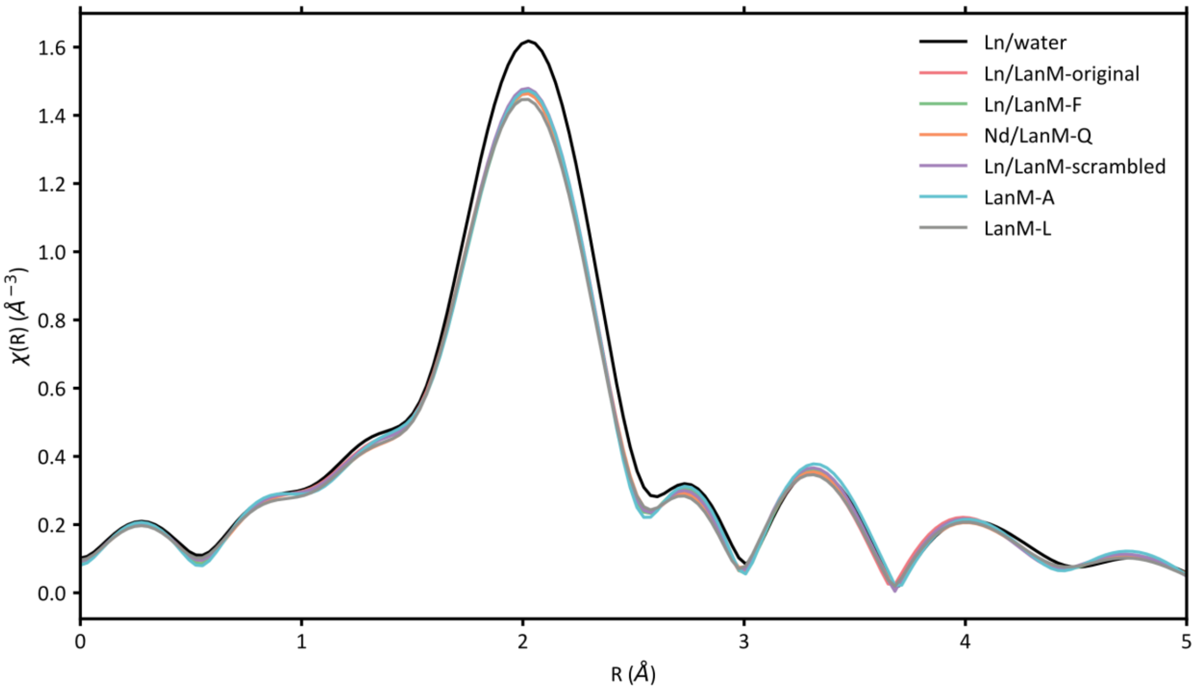

Figure S14 The R-space of water and the six peptide variants complexed with Nd (L2-edge and k-range: 2.53-8.5 Å-1).

S7. Flow Cell

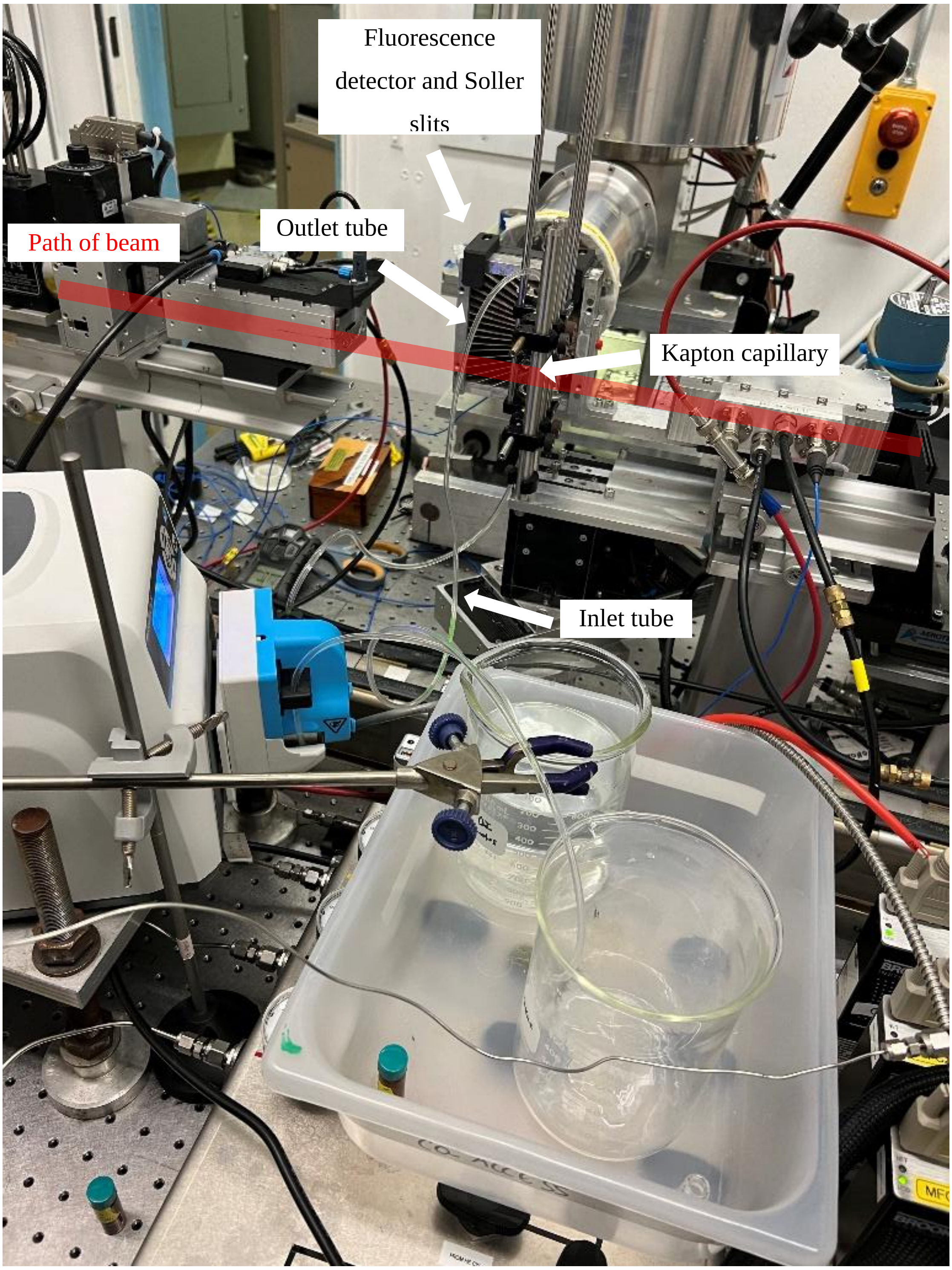

Figure S15 Overview of the entire flow cell setup within the beam line hutch.

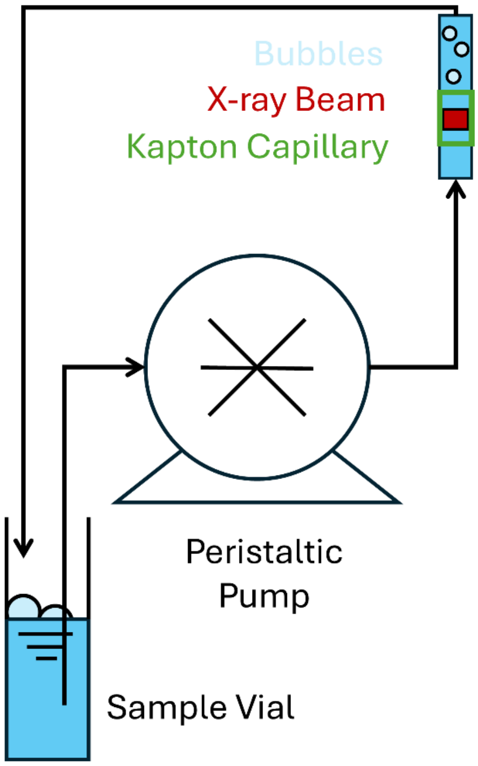

Figure S16 Schematic of the flow cell used in this study. The sample (dark blue) was pumped from a vial using a peristaltic pump through a Kapton capillary (green) where the X-ray beam (red) hit the sample. Bubbles (light blue) generated from the X-ray beam are deposited atop the sample vial and never cross the beam.

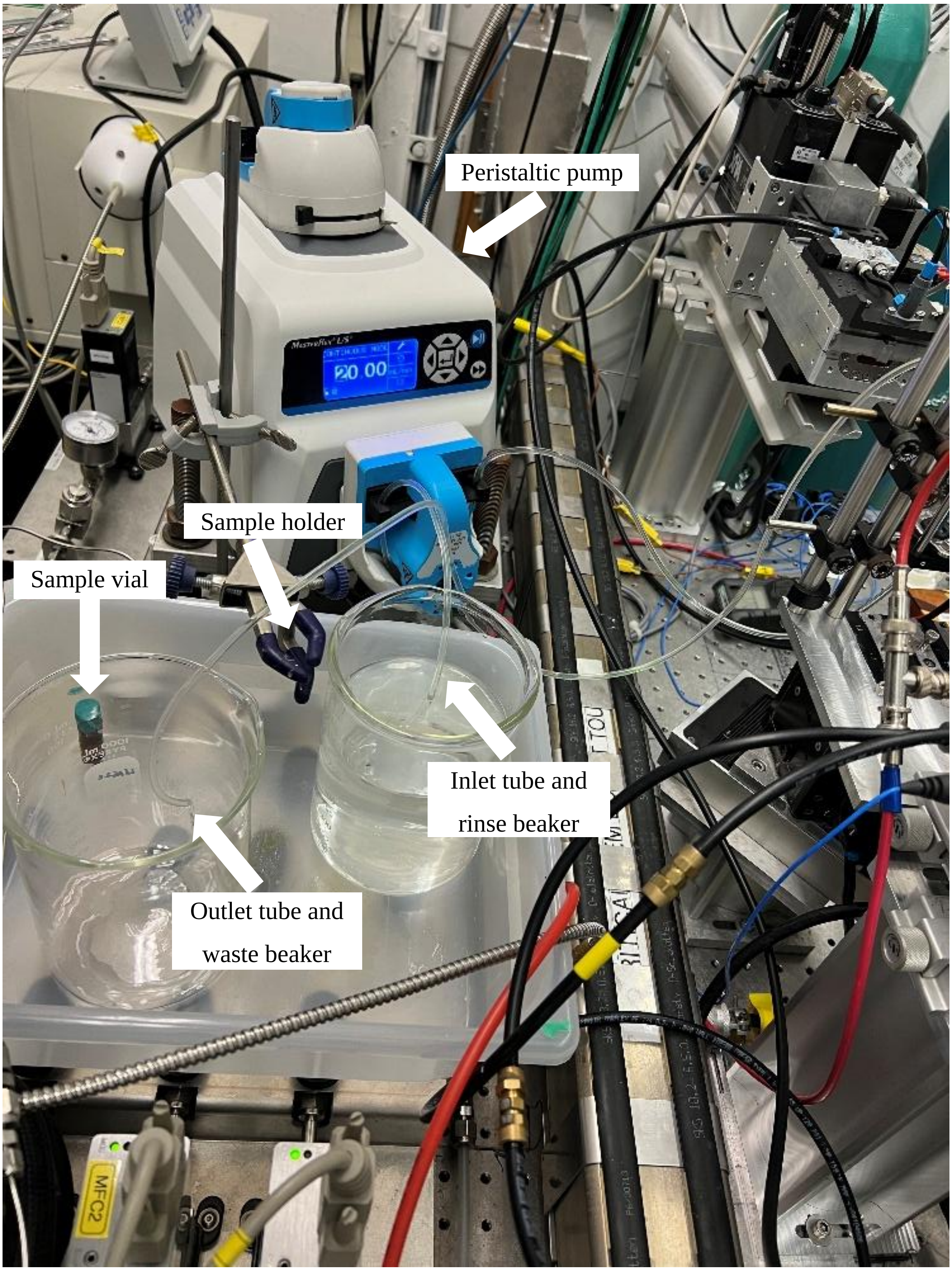

Figure S17 Close-up view of the sample holder, rinse and waste beakers, pump, and tubing during flow cell cleaning. During data acquisition, the sample vial would be in the sample holder and both the inlet and outlet tubing would be in the sample vial.

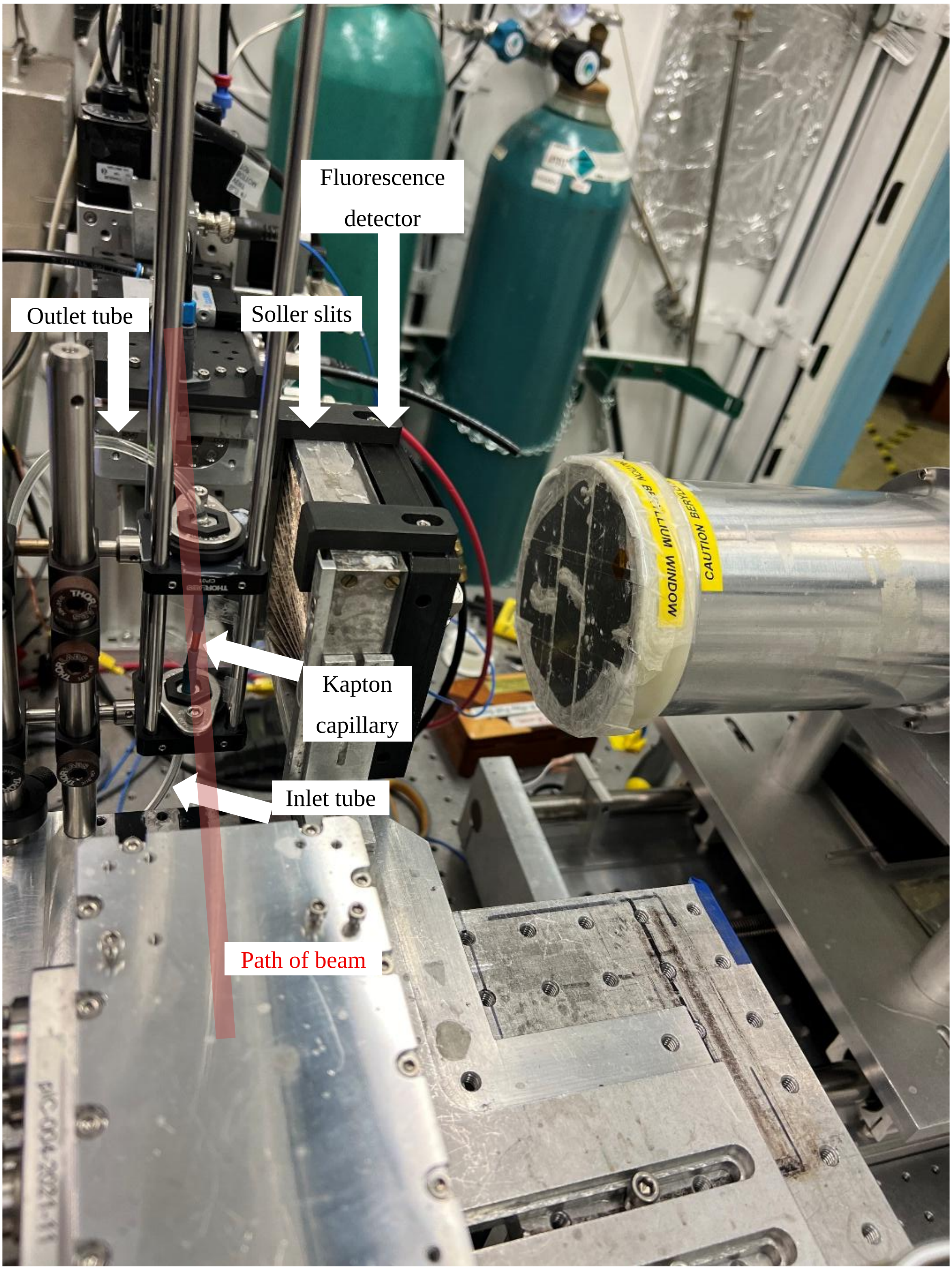

Figure S18 Close-up view of the Kapton capillary and fluorescence detector.

S8. XAS Preprocessing Details

All preprocessing was done using the Athena application from the Demeter package (Ravel & Newville, 2005). All samples were measured on beamline 9-3 at the Stanford Synchrotron Radiation Lightsource under the conditions specified in the main text. Samples were calibrated and aligned according to the main text. Figure S19 shows how the pre- and post-edge lines were chosen to normalize the data. The pre-edge was linear, and the post-edge was third order. Figure S20 shows an example of the Athena input used in preprocessing. For each Ln element, the pre- and post- edge ranges were chosen to best follow the data, and the same range was used for all samples of the same Ln element. The edge energy was assigned using the default Athena methods. Spline ranges were assigned using the default Athena methods. The k-range was selected by taking the first zero crossing in k-space after 2 $Å^{-1}$ and the last zero crossing in the data before the next L-edge.

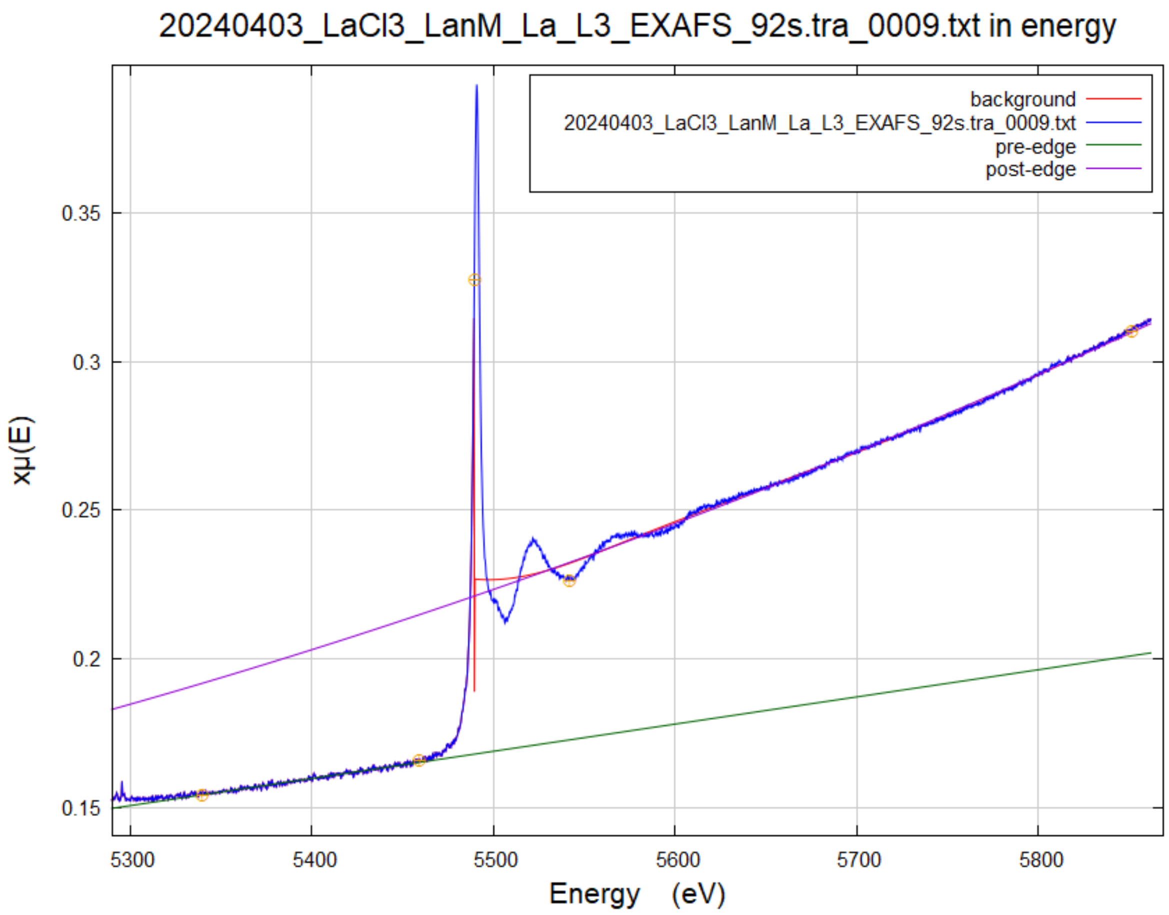

Figure S19 Example of how the pre- and post-edge lines were assigned in Athena during data preprocessing.

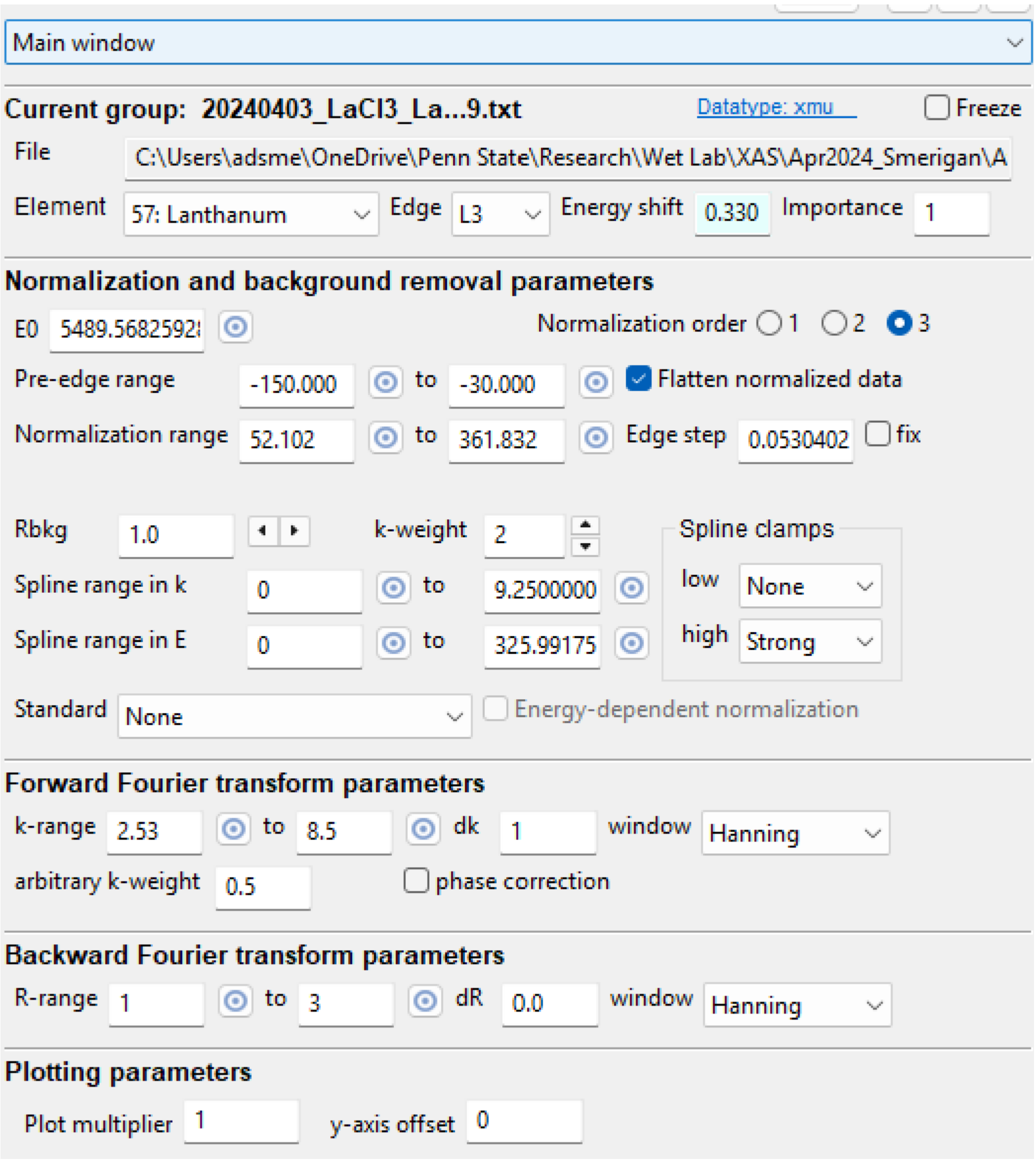

Figure S20 Typical input for Athena for data preprocessing.

S9. R-space spectra from 0-10 Å for Figure 5 and Figure 6c

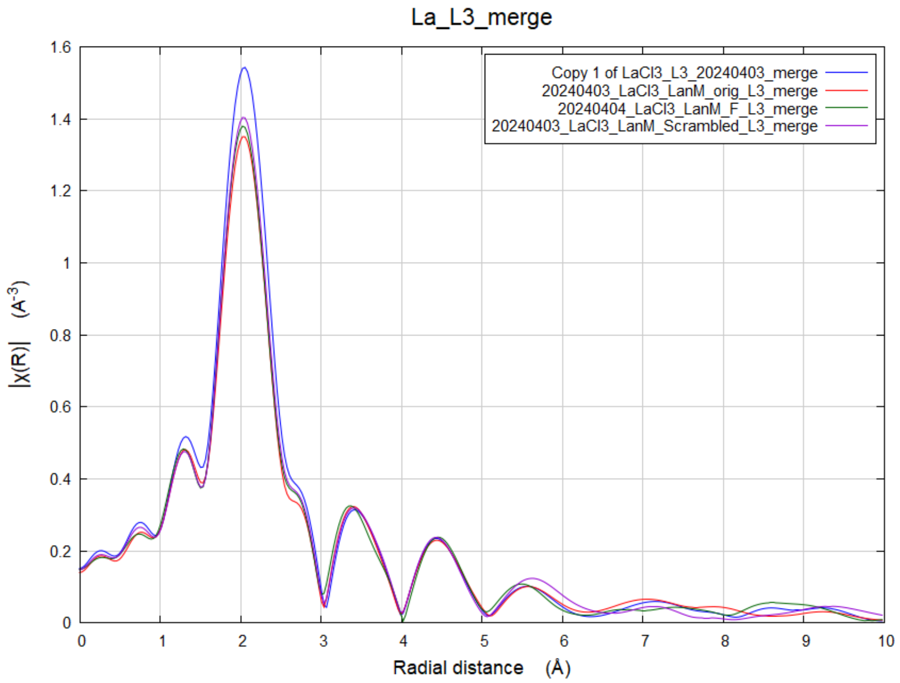

Figure S21 The R-space from 0-10 Å for Figure 5a.

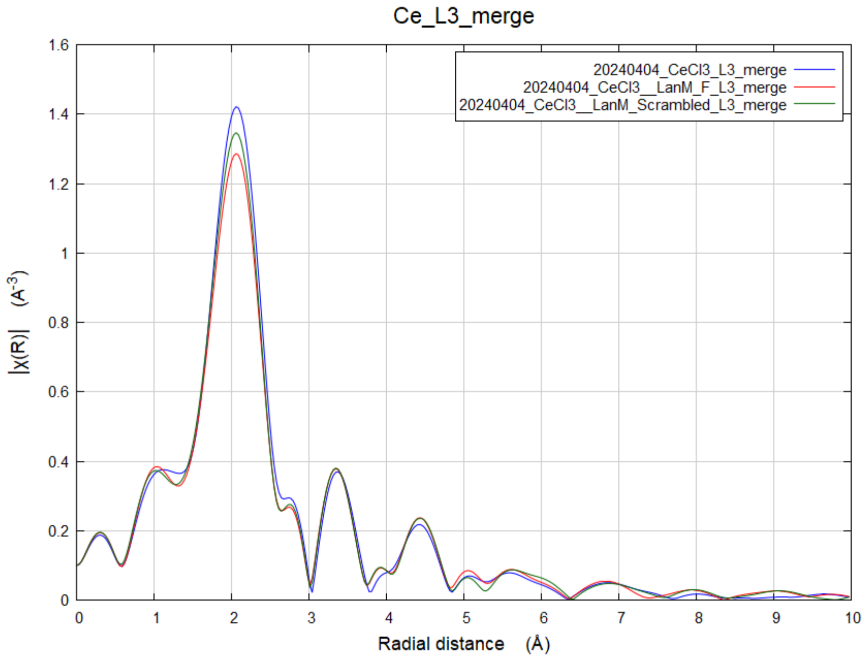

Figure S22 The R-space from 0-10 Å for Figure 5b.

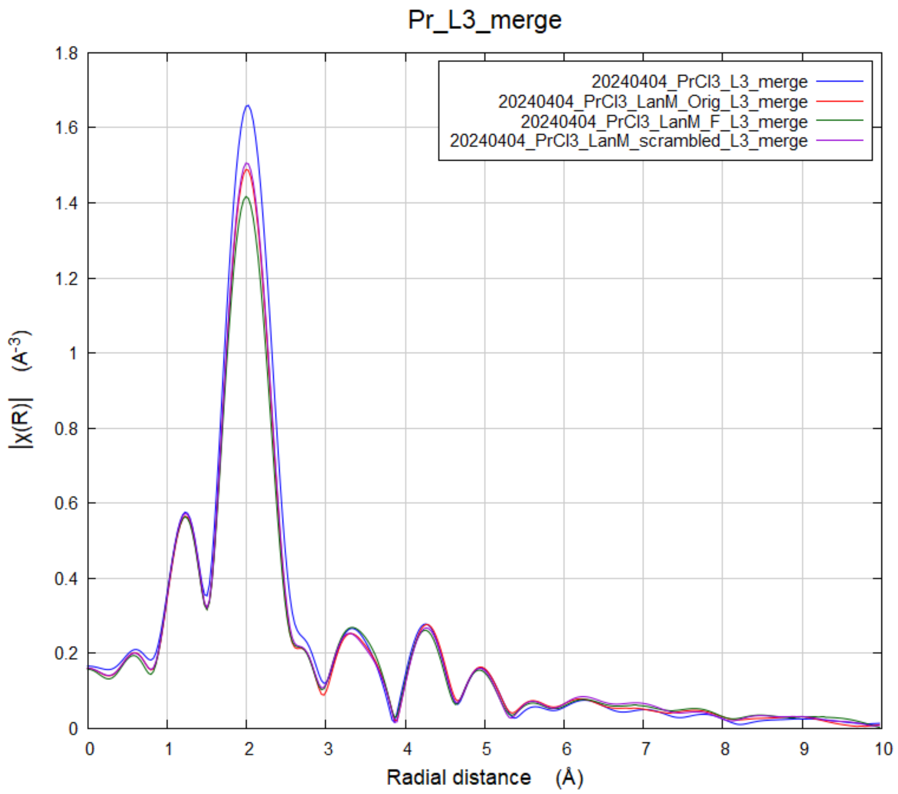

Figure S23 The R-space from 0-10 Å for Figure 5c.

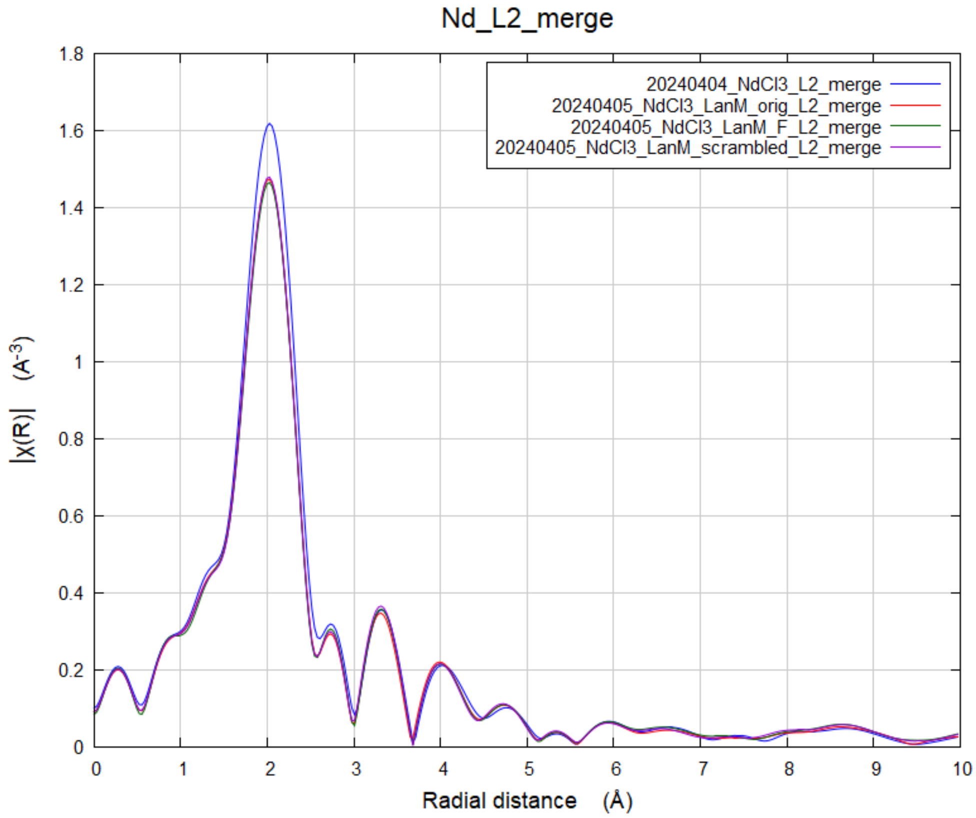

Figure S24 The R-space from 0-10 Å for Figure 5d.

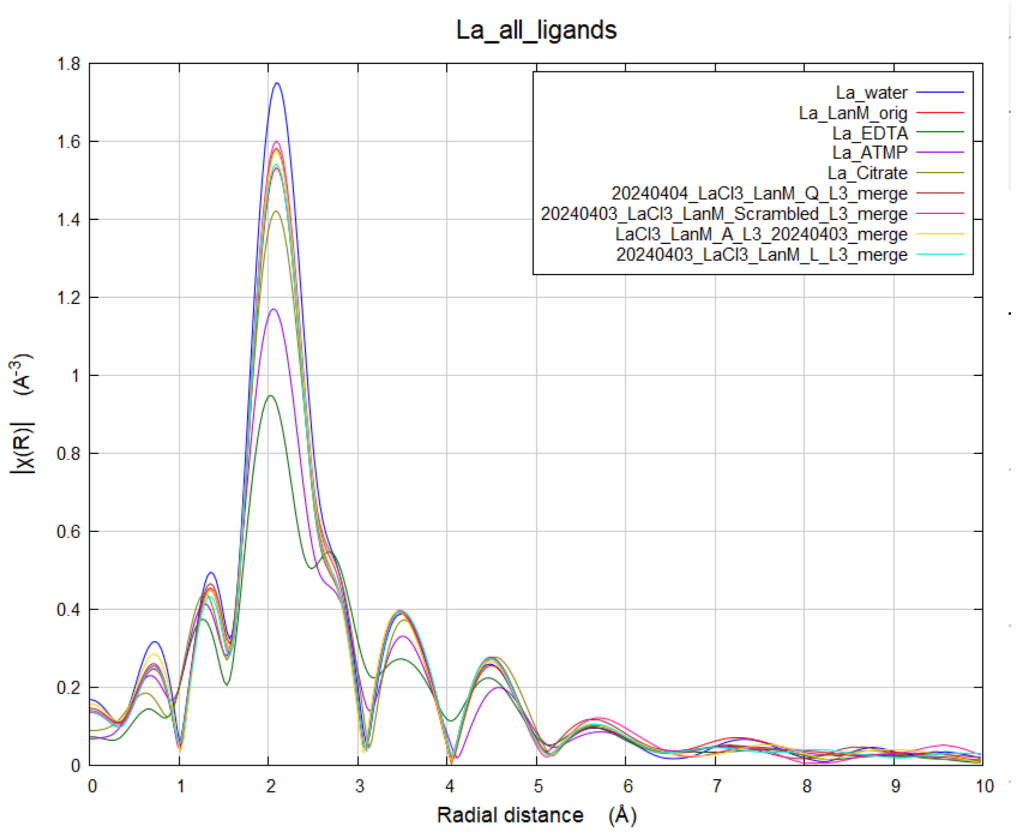

Figure S25 The R-space from 0-10 Å for Figure 6c.

S10. First-shell EXAFS Fit of La/LanM-orig complex

We performed EXAFS modelling using a theoretical structure from a molecular dynamics simulation to confirm complex formation. The EXAFS modelling was performed in the Artemis application from the Demeter package (Ravel & Newville, 2005). The amplitude reduction factor was set to 1 and the coordination number of the scattering paths was set to match the molecular simulation. Only three variables were used in the model: change in edge energy (ΔE_0), change in scattering distance (ΔR), and mean squared radial displacement or Debye Waller factor (MSRD or σ^2). These three free variables are well within the information content of the data of 5.58 independent points. No fuzzy degeneracy was used to condense paths of similar distance. All scattering paths with significant signal were used in the fit and shared the same free variables. Figure S26 shows the measured data in blue and the fit in red. The statistics of the fit are within generally accepted ranges for a good fit: R-factor of 0.017 and a reduced χ^2 of 321 (Calvin, 2013). Further, the value of the free variables remained within physically reasonable ranges: ΔE_0 of 8.9 eV, ΔR of 0.04 Å, and a MSRD of 2.3 (10^{-3} Å^2). From the EXAFS modelling result, the average distance of coordinating oxygen atoms to the La ion is 2.6 Å, which is similar to the average coordinating oxygen distance found in other studies for La/EDTA complexes of 2.58 Å (Smerigan *et al.*, 2023). This EXAFS modelling is preliminary work to confirm complex formation in this study. In the future, more extensive modelling should be performed to determine the exact number, distance, and disorder of coordinating atoms around the Ln ions in solution.

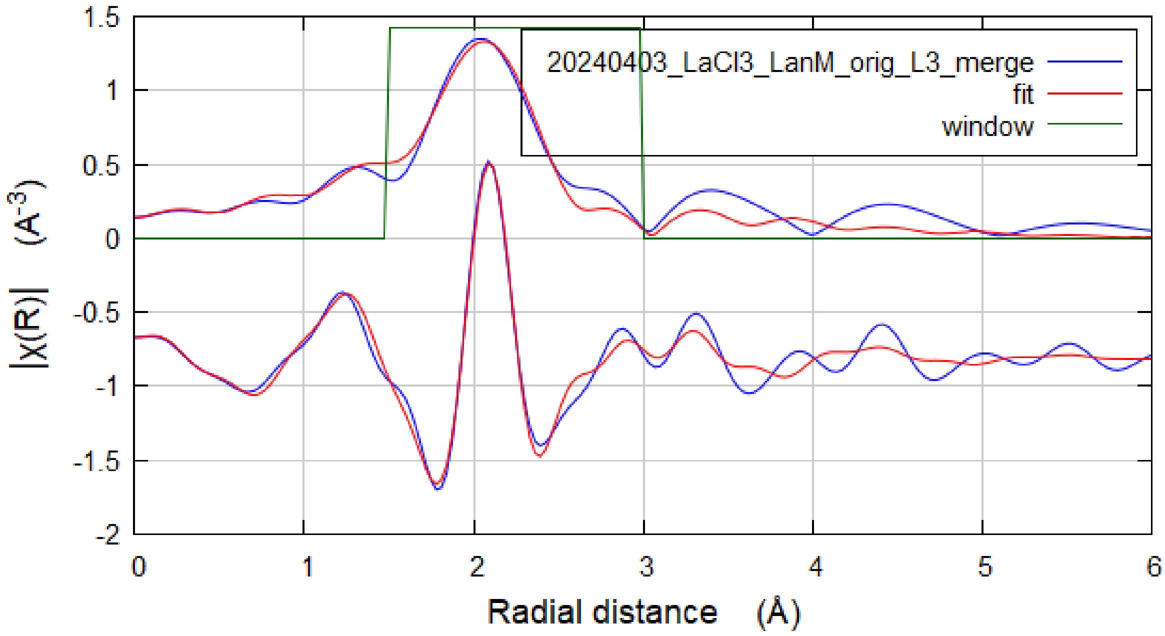

Figure S26 The magnitude of the Fourier transform of the k^2-weighted L_3-edge EXAFS for the La/LanM-orig complex with a k-range of 2.53-8.5 Å^{-1} and R-range of 1.5-3 Å.

S11. References

Calvin, S. (2013). XAFS for Everyone Boca Raton: CRC Press.

Ravel, B. & Newville, M. (2005). *J Synchrotron Rad* **12**, 537–541.

Smerigan, A., Biswas, S., Vila, F. D., Hong, J., Perez-Aguilar, J., Hoffman, A. S., Greenlee, L., Getman, R. B. & Bare, S. R. (2023). *Inorg. Chem.* **62**, 14523–14532.
